# Neural Filtering of Physiological Tremor Oscillations to Spinal Motor Neurons Mediates Short-Term Acquisition of a Skill Learning Task

**DOI:** 10.1523/ENEURO.0043-24.2024

**Published:** 2024-07-17

**Authors:** Hélio V. Cabral, Alessandro Cudicio, Alberto Bonardi, Alessandro Del Vecchio, Luca Falciati, Claudio Orizio, Eduardo Martinez-Valdes, Francesco Negro

**Affiliations:** ^1^Department of Clinical and Experimental Sciences, Università degli Studi di Brescia, Brescia 25123, Italy; ^2^Department Artificial Intelligence in Biomedical Engineering, Friedrich-Alexander University, Erlangen 91052, Germany; ^3^School of Sport, Exercise and Rehabilitation Sciences, College of Life and Environmental Sciences, University of Birmingham, Birmingham B152TT, United Kingdom

**Keywords:** common synaptic input, force control, motor unit, skill learning

## Abstract

The acquisition of a motor skill involves adaptations of spinal and supraspinal pathways to alpha motoneurons. In this study, we estimated the shared synaptic contributions of these pathways to understand the neural mechanisms underlying the short-term acquisition of a new force-matching task. High-density surface electromyography (HDsEMG) was acquired from the first dorsal interosseous (FDI; 7 males and 6 females) and tibialis anterior (TA; 7 males and 4 females) during 15 trials of an isometric force-matching task. For two selected trials (pre- and post-skill acquisition), we decomposed the HDsEMG into motor unit spike trains, tracked motor units between trials, and calculated the mean discharge rate and the coefficient of variation of interspike interval (COV_ISI_). We also quantified the post/pre ratio of motor units’ coherence within delta, alpha, and beta bands. Force-matching improvements were accompanied by increased mean discharge rate and decreased COV_ISI_ for both muscles. Moreover, the area under the curve within alpha band decreased by ∼22% (TA) and ∼13% (FDI), with no delta or beta bands changes. These reductions correlated significantly with increased coupling between force/neural drive and target oscillations. These results suggest that short-term force-matching skill acquisition is mediated by attenuation of physiological tremor oscillations in the shared synaptic inputs. Supported by simulations, a plausible mechanism for alpha band reductions may involve spinal interneuron phase-cancelling descending oscillations. Therefore, during skill learning, the central nervous system acts as a matched filter, adjusting synaptic weights of shared inputs to suppress neural components unrelated to the specific task.

## Significance Statement

Previous studies have proposed that only the low-frequency oscillations of shared synaptic inputs to motor neurons, encompassing task-related and task-unrelated oscillations, are responsible for the generated muscle force. In our study, we investigated whether the acquisition of a new motor task involving precise force generation requires specific alterations in these shared synaptic inputs. Our findings demonstrated that, for both a hand muscle and a leg muscle, the skill acquisition was mediated by a reduction in shared synaptic oscillations unrelated to the required force fluctuations (i.e., physiological tremor band oscillations). Therefore, during the force-matching task learning, the central nervous system acts like a neural filter, modulating the synaptic weights of shared inputs to attenuate neural components unrelated to the specific task.

## Introduction

Over the past three decades, the measurement of incremental motor skill acquisition has emerged as a relevant experimental paradigm for investigating the cognitive and neural processes underlying the learning of new motor abilities (Karni et al., 1998; Ungerleider et al., 2002). Initially, the new movement is produced with varying accuracy, but with repetition and practice, the central nervous system refines the movement, resulting in effortless and precise execution (Willingham, 1998). Consequently, the learning of a new motor task entails structural and functional alterations in the supraspinal and spinal pathways (i.e., neural plasticity) to accommodate the acquisition and retention of skilled motor behaviors (Ungerleider et al., 2002; Dayan and Cohen, 2011). Indeed, compelling evidence from studies on primates (Plautz et al., 1995; Nudo et al., 1996) and nonprimates (Kleim et al., 1998) animals have demonstrated that, following motor skill training, there is an expansion of the cortical representations related to the acquired task. Similar neural adaptations have been observed in humans in studies using noninvasive imaging and neurophysiological techniques (Karni et al., 1995; Pascual-Leone et al., 1995; Perez et al., 2004; Jensen et al., 2005). Specifically, the acquisition of new motor skills has been shown to significantly increase cortical representation (Karni et al., 1995; Pascual-Leone et al., 1995, 1999) and corticospinal excitability (Perez et al., 2004; Jensen et al., 2005) of the muscles involved in the training task. While there is greater consensus regarding the effects of motor skill learning on supraspinal adaptations in humans, the dynamics of plastic changes in synaptic connectivity to spinal motor neurons remain relatively unexplored in the literature.

Many daily activities require the rapid acquisition of motor tasks that demand precise control of fine movements within short time intervals (Johansson and Westling, 1988). As the generation of precise movements relies on the accurate modulation of muscle forces (Johansson and Westling, 1988; Kumar et al., 2017), specific control strategies are necessary during motor skill learning to overcome the inherent variability of the neural pathways innervating muscles. Interestingly, previous research has demonstrated that the motor neuron pool behaves as a very selective spatial filter, eliminating components of synaptic input that are not common to all motor neurons (Negro et al., 2009, 2016b; Negro and Farina, 2011b; Farina et al., 2014). Additionally, it has also been observed that the muscle itself acts as a temporal smoothing filter of the neural drive (Bawa and Stein, 1976; Baldissera et al., 1998), further minimizing the high-frequency components of the synaptic noise across the alpha motor neuron pools. Collectively, these investigations indicate that only the low-frequency components of the synaptic inputs widely shared across the motor neuron pools are represented in the force output (Enoka and Farina, 2021). In voluntary tasks, these shared synaptic inputs consist of task-related oscillations, which determine the precise command for optimal force generation (e.g., control input signals from corticospinal pathways), as well as task-unrelated oscillations. These task-unrelated oscillations comprise both voluntary components (reflecting errors in task performance) and involuntary components (e.g., physiological tremor) that act as noise, thereby reducing the precision of the task (Bays and Wolpert, 2007). Thus, the process of learning new motor tasks involving precise force generation should require minimizing these task-unrelated oscillations, whether voluntary or involuntary, to maximize the representation of shared synaptic input related to optimal control in the force output (i.e., task-related oscillations; Harris and Wolpert, 1998; Bays and Wolpert, 2007).

Despite extensive research exploring the effect of motor learning on reducing errors in task performance (Ungerleider et al., 2002; Dayan and Cohen, 2011), no study has yet experimentally investigated whether the acquisition of a new motor skill involves a reduction in the involuntary components of the shared synaptic input. Particularly intriguing is the observation that during steady contractions, the force output exhibits involuntary oscillations ∼10 Hz (alpha band), commonly associated with physiological tremor (McAuley and Marsden, 2000). The origin of these rhythmic fluctuations has been attributed to cortical pathways (Marsden et al., 1967; McAuley et al., 1997; Evans and Baker, 2003) and/or peripheral pathways, likely originating from the Ia afferent feedback loop (Halliday and Redfearn, 1956; Lippold, 1971). If indeed the acquisition of new motor skills is mediated by reductions in physiological tremor, specific cortical and/or peripheral neural mechanisms must emerge during the skill learning task to reduce alpha band oscillations in the shared synaptic input to motor neurons. For instance, previous data in macaque monkeys have shown that spinal interneurons could phase-cancel ∼10 Hz cortical inputs to motor neurons, which would be beneficial to decrease force tremor and improve movement precision (Williams et al., 2010; Koželj and Baker, 2014). Therefore, it is possible that during motor skill learning, the gain of this spinal interneurons filter could be upregulated, thereby increasing the cancellation. On the other hand, compelling evidence has demonstrated that changes in the gain of afferent feedback loop may directly modulate physiological tremor inputs (Cresswell and Löscher, 2000; Christakos et al., 2006; Laine et al., 2016), presenting another potential mechanism that could be involved in the acquisition of a skill learning task. Even though both mechanisms appear intuitively reasonable, no study to date has explored whether either of these mechanisms plays a role during the acquisition of a skill learning task.

The present study aimed to investigate whether the short-term acquisition of a new force-matching skill is mediated by alterations in the shared synaptic input to spinal motor neurons, particularly in the physiological tremor band (alpha band; 5–15 Hz). We hypothesized that the acquisition of a new motor skill in an individual muscle would require specific adaptations in the neural pathways to the motor neuron pool of that muscle, ultimately reducing the contributions of shared synaptic oscillations unrelated to the task (i.e., alpha band oscillations). We experimentally tested this hypothesis by decomposing high-density surface electromyograms from the first dorsal interosseous (FDI) and tibialis anterior (TA) muscles during 15 trials of a complex, isometric force-matching task (force-matching skill acquisition). In addition, we simulated a population of motor neurons receiving both common and independent inputs to elucidate the potential neural mechanisms underlying the experimental results. Specifically, we simulated two scenarios: filtering of alpha oscillations by spinal interneuron circuits (Scenario A) and filtering of alpha oscillations in the Ia afferent feedback loop (Scenario B). We then compared the results of the two scenarios with the experimental results, aiming to determine which scenario best explains the observed experimental outcomes.

## Materials and Methods

### Participants

Twenty-four healthy volunteers participated in the study. Specifically, two experiments were performed in which 13 participants (6 females; age, 31 ± 4 years; height, 176.8 ± 7.1 cm; mass, 71.9 ± 16.4 kg) performed the FDI muscle protocol, and 11 (4 females; age, 31 ± 3 years; height, 174.9 ± 9.0 cm; mass, 71.2 ± 18.1 kg) performed the TA muscle protocol. All participants were free from musculoskeletal or neurological injuries and provided written informed consent prior to the beginning of experiments. This study was approved by the local ethics committee (code NP5665) and conformed to the latest Declaration of Helsinki.

### Experimental design

**Figure 1.**
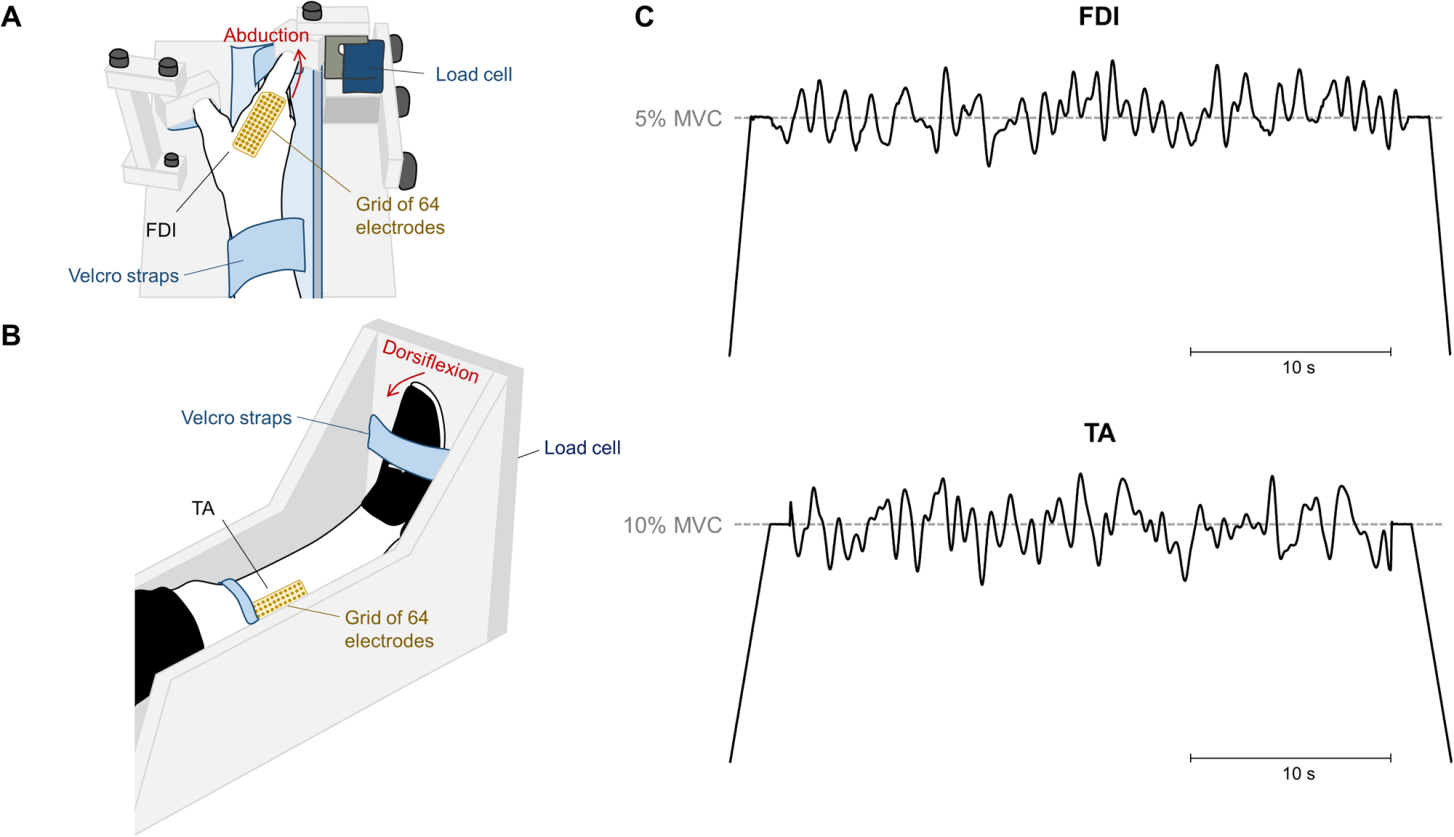
Experimental setup. ***A***, Position of the participants’ wrist and hand on the custom-made dynamometer to record the abduction isometric force produced by the index finger. ***B***, Position of the participants’ shank and foot on the custom-built dynamometer to measure the dorsiflexion isometric force produced by the ankle. High-density surface electromyography grids were placed over the first dorsal interosseous (FDI) muscle (***A***) and tibialis anterior (TA) muscle (***B***). ***C***, Isometric force-matching tasks presented to the participants for the FDI (top) and the TA (bottom) muscles. Tasks involved a plateau region of 30 s containing a randomly generated signal low-pass filtered at 1.5 Hz. The target force level was defined as 5 and 10% of MVC for the FDI and TA, respectively. The same trajectory was used throughout all trials of the force-matching skill task.

Participants took part in a single experimental session lasting ∼1 h. For the measurements on the FDI muscle, participants sat on an adjustable chair with their elbow flexed at 45° (0° being the anatomical position) and their right upper arm and hand comfortably resting on a custom-made device. The wrist and the hand were in a neutral position. The middle phalanx of the index finger was fixed to an adjustable support attached to a load cell (SM-100 N, Interface) so that the isometric abduction force produced by the index finger could be measured (Fig. 1*A*). To standardize the hand position and minimize the contribution of other muscles, the little, ring, and middle fingers were separated from the index finger and secured to the device with Velcro straps. The forearm was also strapped to the device, and the thumb was secured at ∼80° angle to the index finger (Fig. 1*A*). For the measurements on the TA muscle, participants were comfortably seated on a custom-built ankle dynamometer with their right knee fully extended, their ankle at 10° of plantar flexion (0° being the foot perpendicular to the shank), and their hip flexed at 70° (0° being the hip fully extended). The right foot was fixed with Velcro straps to an adjustable footplate perpendicularly connected to a load cell (SM-500 N, Interface) to record the dorsiflexion isometric force produced by the ankle (Fig. 1*B*).

For both TA and FDI muscles, participants were initially asked to perform three isometric maximal voluntary contractions (MVCs) for 3 s, with a 60 s interval of rest in between. The greatest value across the three MVCs was considered the maximal isometric force and used as a reference for the following submaximal contractions. Then, after 5 min rest period, participants were instructed to perform 15 trials of a complex, isometric force-matching task (force-matching skill acquisition). The task involved a linear increase in force at a rate of 5% MVC/s, a variable force region for 30 s, and a linear decrease in force at a rate of 5% MVC/s. The variable force region contained oscillations above and below the target force level (averaged exerted force), which was defined as 5% MVC for the FDI and 10% MVC for the TA. The level of 5% MVC was chosen for the FDI to minimize fatigue effects across the task. Specifically, the oscillations consisted of a randomly generated signal with frequency content below 1.5 Hz (−3 dB low-pass frequency). Two trajectories were generated, one for each muscle (Fig. 1*C*), and the same trajectory was used throughout all trials and for all the subjects (Knight and Kamen, 2004). Each trajectory consisted of a black line, depicting the target force, over a white background and a red line depicting the subject's force. A minimum of 60 s of rest was provided between trials, and prior to each trial, participants were encouraged by the same investigator to follow the target as closely as possible. Visual feedback from the target and the force was displayed on a computer monitor positioned at ∼60 cm in front of the participant, in which the entire target trajectory was visible and stationary.

To ensure that the changes in shared synaptic oscillations within alpha band observed with the force-matching skill acquisition (see Results) were not attributable to the sole motor execution, independent of learning, we implemented a control condition involving a subgroup of three participants (3 males; mean ± SD: age, 36 ± 10 years; height, 181 ± 6 cm; mass, 82 ± 25 kg). In these experiments, participants underwent testing on their TA muscle following a protocol similar to the one previously described. However, instead of engaging in 15 trials of a complex force-matching task, they were instructed to perform 15 trials of steady isometric contractions at 10% MVC. Each trial lasted 30 s with a minimum of 60 s of rest between trials. To prevent learning effects, participants were presented with a square indicating the target force level, and they were instructed to move a pointer, which was proportional to the real-time generated force, inside this square to match the target force.

### Data collection

During all trials of the force-matching task, high-density surface electromyograms (HDsEMG) signals were acquired from FDI and TA muscles using a grid of 64 electrodes arranged into 13 rows × 5 columns, with a missing electrode on a corner (4 mm interelectrode distance for FDI; 8 mm interelectrode distance for TA; OT Bioelettronica). An experienced investigator determined the position and orientation of the grids via palpation of anatomical landmarks. Specifically, the electrodes were attached over the belly of each muscle in the following locations: FDI, lateral to the line connecting the heads of the first and second metacarpals (Fig. 1*A*), and TA, ∼1 cm lateral to the tibial prominence (Fig. 1*B*). Electrodes were fixed to the skin using a bi-adhesive foam, and the electrode-skin contact was ensured by filling the foam cavities with conductive paste (AC cream, Spes Medica). Reference electrodes were positioned on the right wrist for the FDI and on the right ankle for TA. Prior to electrode placement, the skin was cleaned with an abrasive paste (EVERI, Spes Medica) and shaved when necessary. Surface EMGs were recorded in monopolar mode and digitized at 2,048 samples/s using a 16 bit amplifier (10–500 Hz bandwidth; Quattrocento, OT Bioelettronica). Force signals provided by the load cell were amplified by a factor of 100 (Forza-j, OT Bioelettronica) and sampled synchronously with EMGs.

### Data analysis

Force and HDsEMG signals were analyzed offline using MATLAB custom-written scripts.

#### Force

First, force signals were low-pass filtered at 15 Hz using a third-order Butterworth filter. Then, to quantify the performance for each trial of the force-matching task, the root-mean-square error (RMSE) between the force and target signals was computed for the middle 30 s of the target (i.e., oscillatory region; Knight and Kamen, 2004). Cross-correlation peak values were also calculated for the middle 30 s to assess similarities between fluctuations in force and target signals. For both RMSE and cross-correlation analyses, the detrended version of the signals was used. To assess force-matching improvements during the skill acquisition task, the first two and last two trials (out of the 15 trials) were selected for each participant. Since there were no statistical differences in RMSE and cross-correlation within both the initial two trials and the final two trials (see Results), we chose to select one trial from the first two and one trial from the last two to represent the pre- and post-skill acquisition trials for all subsequent analysis. This decision was also driven by the aim to maximize the number of tracked motor units between trials (see below, Identification and tracking of motor units). Specifically, the trials selected were the ones with the highest and smallest RMSE between the force and target signals. To quantify how force steadiness changed during the learning task, we calculated the coefficient of variation (i.e., standard deviation/mean) of detrended force signals in the two selected trials. In addition, the power spectral density of force signals from the pre- and post-skill acquisition trials was estimated using Welch's method (*pwelch* function in MATLAB; 1 s Hanning windows with 95% of overlap). For each trial, the mean force power within the delta (1–5 Hz) and alpha (5–15 Hz) bands was calculated and retained for further analysis. Only the delta and alpha bands were considered in this analysis because the frequency range of force signals was up to 15 Hz.

#### Identification and tracking of motor units

**Figure 2.**
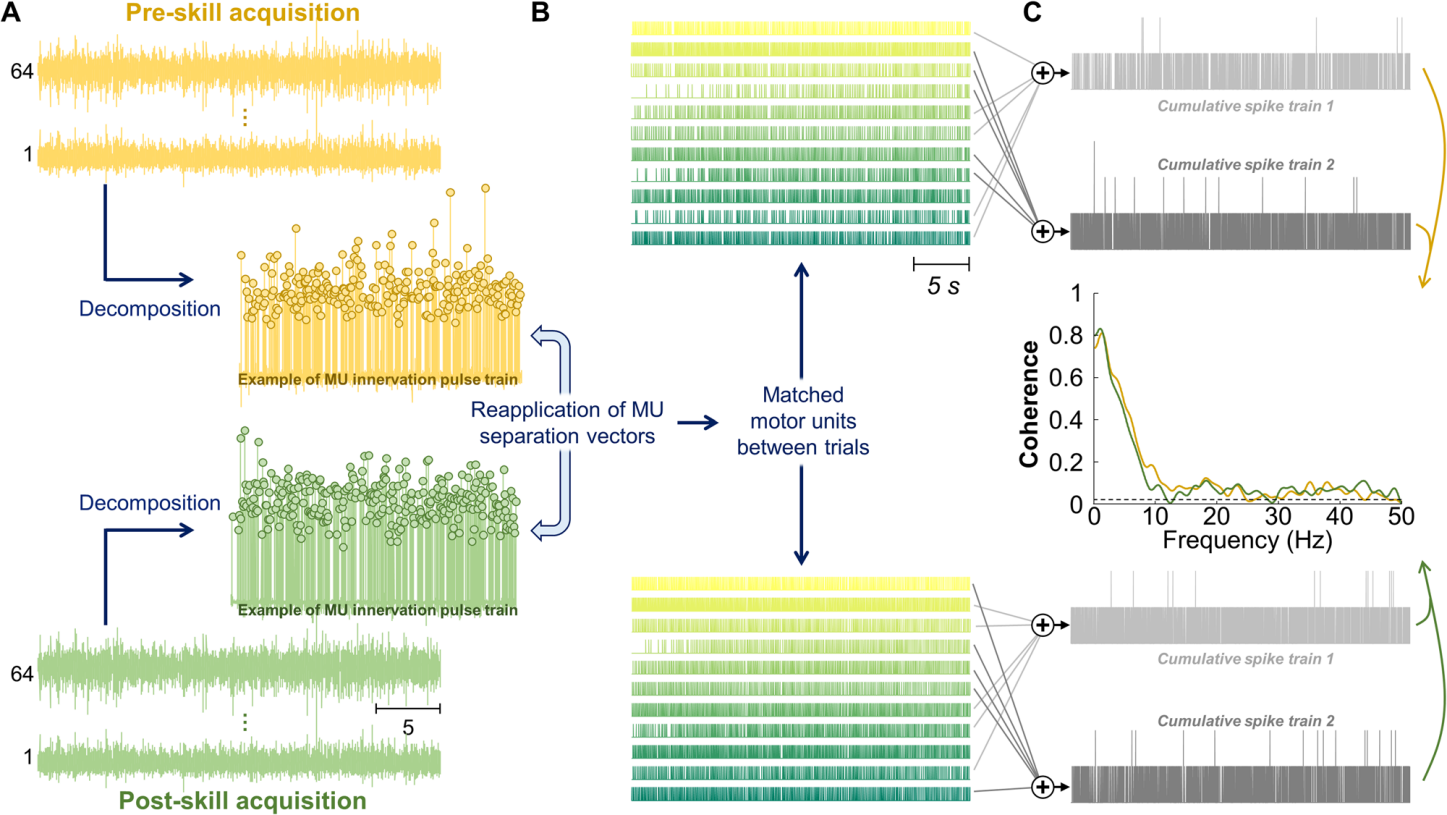
Method for common synaptic input estimation. ***A***, Motor unit identification and tracking via decomposition from high-density surface electromyography signals, with motor unit separation vectors reapplied between trials. ***B***, Raster plots of matched units between trials. ***C***, Estimation of common synaptic oscillations using coherence analysis. This involved analyzing two equally sized CSTs, derived by summing the discharge times of motor units randomly selected from matched motor units.

Similar to the force, all motor unit analyses were performed for the middle 30 s of the target (i.e., oscillatory region). For the experimental group, HDsEMG signals acquired during the pre- and post-skill acquisition trials were used. For the control group, data from the first and last trials were used. First, HDsEMG signals were bandpass filtered with a third-order Butterworth filter (20–500 Hz cutoff frequencies). After visual inspection, channels with low signal-to-noise ratio or artifacts were discarded. Then, the HDsEMG signals were decomposed into motor unit spike trains using a convolutive blind source separation algorithm (Negro et al., 2016a; Fig. 2*A*). This method has been previously validated and extensively applied to assess the activity of single motor units (Castronovo et al., 2015; Negro et al., 2016a; Cogliati et al., 2020; Hassan et al., 2020). After the automatic identification of motor units, all the motor unit spike trains were visually inspected for false positives or false negatives (Hassan et al., 2020). Missing pulses or incorrectly assigned pulses producing nonphysiological discharge rates were manually and iteratively edited by an experienced operator, and motor unit spike trains were re-estimated as previously proposed (Martinez-Valdes et al., 2017; Hassan et al., 2020). This approach has been shown to be highly reliable across operators (Hug et al., 2021). After the editing of motor unit spike trains, the motor units were tracked between the pre- and post-skill acquisition trials. This was achieved by reapplying the motor unit separation vectors, which are estimated with the blind source separation algorithm, from one trial to the other (Oliveira and Negro, 2021; Rossato et al., 2022; Fig. 2*B*). These motor unit separation vectors are unique for each individual motor unit and define the spatiotemporal matched filters to estimate the motor unit spike trains. This tracking procedure was performed in the forward and backward directions (i.e., motor unit separation vectors of pre-skill acquisition trial were applied on the post-skill acquisition trial and vice versa). Thus, our approach ensured that the same motor units were tracked and analyzed in both trials. Figure 2*B* illustrates an example of spike trains from motor units tracked between trials. Only motor units spike trains with a silhouette value, which is a metric to assess decomposition accuracy (Negro et al., 2016a), higher than 0.85 were used for analysis. For three participants in FDI and one participant in TA, we tracked the motor units based on their shape (Martinez-Valdes et al., 2017), as we were not able to track at least four motor units (see below, Estimates of common synaptic input) by reapplying the motor unit separation vectors. For this analysis, the two-dimensional representations of the motor unit action potentials of the identified motor units in the pre-skill acquisition trial were cross-correlated with the two-dimensional representations of the identified motor units in the post-skill acquisition trial (Martinez-Valdes et al., 2017; Cogliati et al., 2020). Only motor units with highly similar motor unit action potentials (cross-correlation >0.8) were considered as belonging to the same motor units. The mean discharge rate and coefficient of variation of interspike interval (COV_ISI_) were calculated for each matched motor unit and stored for further analysis.

#### Estimates of common synaptic input

To assess changes in common synaptic input to motor neurons with the force-matching skill acquisition (experimental group) and repeated motor execution (control group), coherence analysis was performed between motor units from the same muscle (Negro and Farina, 2012; Castronovo et al., 2015; Negro et al., 2016b; Maillet et al., 2022; Rossato et al., 2022). Coherence is a frequency-domain linear coupling measure between two signals. This analysis was performed on two equally sized cumulative spike trains (CSTs), which were obtained by summing the binary discharge trains of motor units randomly selected from the identified, matched units (Fig. 2*C*). The number of motor units selected for each of the two groups was half of the total number of detected units, and this was repeated for up to 100 permutations. Only participants with at least four matched motor units were included in the coherence analysis. For all permutations, coherence was calculated between the two detrended CSTs using Welch's periodogram with a 1 s Hanning window and an overlap of 95%. The obtained values were averaged for all permutations and transformed into standard *z*-scores as described by Gallet and Julien (2011). Only *z*-scores greater than the bias were considered for further analysis. The bias was determined as the mean value of *z*-scores between 250 and 500 Hz, as no significant coherence is expected in this frequency range (Castronovo et al., 2015; Maillet et al., 2022). To evaluate changes between trials, the areas under the curve of the *z*-coherence profiles within the delta (1–5 Hz), alpha (5–15 Hz), and beta (15–35 Hz) bands were calculated, and then, the area under the curve ratio (post/pre) was computed separately for each bandwidth. We subtracted one from the values of the area under the curve ratio so that values higher and lower than 0 indicated, respectively, an increase and decrease in *z*-coherence during post- compared with pre-skill acquisition trial. For purposes of visualization only, the pooled *z*-coherence across all participants was calculated as previously proposed (Baker et al., 2003).

Considering the nonlinear relation between the synaptic input to motor neuron and its output spike train becomes more accentuated at higher frequencies (due to the slow operating frequency of individual motor neurons; i.e., ∼30–40 pps), a greater number of motor units are required to accurately estimate coherence within the beta band (Farina and Negro, 2015). As detailed in the Results section, no significant differences were observed in *z*-coherence within this band between pre- and post-skill acquisition trials for both TA and FDI muscles. Therefore, to ensure that the absence of differences within this frequency band was not due to the number of motor units utilized in the CST, we conducted an additional analysis using the TA motor unit data (muscle with greater number of motor units matched between trials). Specifically, we calculated *z*-coherence between two CSTs, as described previously, but increasing the number of motor units in each CST incrementally from 1 to 13 (half of the maximal number of matched motor units across all participants). Subsequently, for each condition (1 motor unit in each CST, 2 motor units in each CST, and so forth), we computed the area under the curve ratio (post/pre) of *z*-coherence within the beta band. This process generated a curve depicting the *z*-coherence ratio within beta band as a function of the number of motor unit spike trains used in the CST. If our results within the beta band were influenced by the number of motor units used in the CSTs, we would expect to observe a significant deviation from zero in this curve as the number of motor units increased. Of note, in cases where a participant could not reach 13 motor units in each CST due to a lower number of identified units, we estimated the area under the curve of *z*-coherence within beta band by linear interpolation, using the other area under the curve values for that participant as reference points.

#### Coherence between force/neural drive and target template

To evaluate changes in the coupling between oscillations in force/neural drive and oscillations in the target template, we calculated the coherence between the force and the target template and between the neural drive to muscles and the target template. In both cases, an increase in coherence values between pre- and post-skill acquisition trials would indicate an enhancement in the representation of shared synaptic input oscillations relevant to optimal force control in the intended motor task (i.e., task-related oscillations). For this analysis, the neural drive to the muscles was estimated by summing the binary discharge trains across all identified motor units (i.e., CST; Thompson et al., 2018). Moreover, coherence was calculated only within the frequency bandwidth of the target template (i.e., delta band). Similar to motor unit analyses, the area under the curve ratio (post/pre) of *z*-coherence was computed to assess changes between pre- and post-skill acquisition trials.

### Simulations

To elucidate the neural mechanisms underlying the observed reductions in alpha band oscillations with the acquisition of the force-matching skill (see Results), we simulated the sequence of events from the excitation of an ensemble of motor neurons to the generation of isometric force output using the model proposed by Fuglevand et al. (1993). In this model, the population of motor neurons received common and independent inputs in varying relative proportions (Negro and Farina, 2012). Detailed descriptions of the modeling approach can be found in previous studies (Negro and Farina, 2011a; Farina et al., 2014; Negro et al., 2016b; Dideriksen and Negro, 2018). The motor neuron parameters were consistent with those used by Cisi and Kohn (2008) and were selected according to an exponential distribution over the pool of motor neurons (Fuglevand et al., 1993).

The number of motor neurons was set to 450 (similar to the number of TA motor units; Feinstein et al. 1955), with only those having a minimum discharge rate of 8 pulses per second (pps) being fully recruited (Taylor et al., 2002). The input to the motor neuron pool was modeled as a linear summation of common synaptic input to all motor neurons and an independent noise input specific to each motor neuron (Negro et al., 2016b). The common synaptic input, which represents the input originating from the brainstem, spinal interneurons or muscle afferents, included both task-related and task-unrelated oscillations. In our simulations, the task-related oscillations (i.e., oscillations within the delta band) were simulated using the same random signal provided as the target to participants during the skill acquisition task in the TA experimental recordings. Task-unrelated oscillations were simulated as the linear summation of 5–15 Hz Gaussian noise (i.e., oscillations within the alpha band) and 15–60 Hz Gaussian noise (i.e., oscillations within beta and piper bands). To explore the effects of afferent feedback modulation, we also included in the model a presynaptic gain of Ia afferent feedback into the motor neuron pool. Finally, the independent noise input, representing the individual variability of the membrane potential of each motor neuron, was modeled as a Gaussian noise with a bandwidth of 50 Hz (Negro et al., 2016b).

Two different scenarios were simulated to investigate the neural mechanisms underlying the observed changes between pre- and post-skill acquisition (refer to Fig. 9 in Results). In Scenario A, we hypothesized that decreases in alpha band with the acquisition of the force-matching skill could be explained by spinal interneurons phase-cancelling central oscillatory inputs in the alpha frequency range (Williams et al., 2010; Koželj and Baker, 2014). The gain of this spinal interneurons filter would be upregulated during the skill acquisition task, thereby reducing alpha band oscillations between pre- and post-skill acquisition. To simulate this scenario, we created two models to represent pre- and post-skill acquisition. Then, we simulated an increase in the gain of the spinal interneurons filter in the post-skill acquisition model by decreasing the standard deviation of the 5–15 Hz input to the motor neuron pool compared with the pre-skill acquisition model. In Scenario B, we hypothesized that reductions in alpha band with the force-matching skill acquisition could be explained by increases in presynaptic inhibition of Ia afferent feedback into the motor neuron pool. Similarly, we created two models to represent pre- and post-skill acquisition, but in this case, we simulated an increase in presynaptic inhibition of Ia afferent feedback in the post-skill acquisition model by decreasing the gain of the Ia afferent input to the motor neuron pool. In both scenarios, each model (pre- and post-skill acquisition) was repeated 10 times as has been done in previous stimulation studies (Dideriksen and Negro, 2018). Following the same approach on the experimental data, we calculated the simulated force output power spectrum; the ratio (post/pre) of the area under the curve of motor unit *z*-coherence within delta (1–5 Hz), alpha (5–15 Hz), and beta (15–35 Hz) bands; and the area under the curve ratio (post/pre) of *z*-coherence between simulated force/CST and the target template. We then compared the results of Scenarios A and B with the experimental results to explore which simulated scenario aligns more closely with the observed experimental outcomes.

### Statistical analysis

All statistical analyses were performed in R (version 4.3.0), using RStudio environment (version 2023.03.1).

To compare the RMSE between the four selected trials (first two and last two), Friedman tests were used. When significant effect of “trial” was detected, post hoc tests with Bonferroni’s correction were conducted for pairwise comparisons. To compare the coefficient of variation of force, and mean force power within delta and alpha bands between pre- and post-skill acquisition trials, Wilcoxon signed-rank tests were used. The Wilcoxon signed-rank test was also used to compare mean force power within delta and alpha bands between the pre-skill acquisition and post-skill acquisition models.

To compare the mean discharge rate and COV_ISI_ between pre- and post-skill acquisition trials, we applied linear mixed-effect models, as they allow for the inclusion of all detected units and not just the mean value for each participant and trial (Boccia et al., 2019). This statistical model accounts for the nonindependence of observations, which is particularly useful for this experimental design due to the hierarchical nature of motor unit data (greater correlation for units within participants compared with between participants; Tenan et al., 2014). For both mean discharge rate and COV_ISI_, random intercept models were applied with “trial” (pre- and post-skill acquisition) as fixed effect and “participant” as random effect [e.g., mean discharge rate ∼1 + trial + (1 | participant)]. LMMs were implemented using the package *lmerTest* (Kuznetsova et al., 2017) with the Kenward–Roger method to approximate the degrees of freedom and estimate the *p* values. The *emmeans* package was used to determine estimated marginal means and their differences with 95% confidence intervals (Lenth et al., 2019).

For both experimental and simulated data, to compare estimates of common synaptic input (i.e., *z*-coherence) between pre- and post-skill acquisition trials, changes in area under the curve ratio (post/pre) were tested using one-sample Wilcoxon signed-rank test (null hypothesis *µ*_0_ = 0), separately for delta, alpha, and beta bands. For the control group, we conducted descriptive analysis on changes in the area under the curve within the alpha band to compare with those observed in the experimental group. To investigate whether the coherence results within the beta band were influenced by the number of motor units used in the CSTs, we utilized the one-sample Statistical Parametric Mapping test (Pataky et al., 2013). This test allowed us to determine whether the curve of the *z*-coherence ratio within beta band, as a function of the number of motor units (see above, Estimates of common synaptic input), showed a statistically significant deviation from 0. Conceptually, the one-sample Statistical Parametric Mapping test resembles the one-sample *t* test, but it evaluates the entire curve. For both experimental and simulated data, changes in area under the curve ratio of target–force *z*-coherence and target–CST *z*-coherence between pre- and post-skill acquisition trials were assessed using the one-sample Wilcoxon signed-rank test (null hypothesis *µ*_0_ = 0).

For the experimental data, repeated-measures correlations were performed to test whether changes in the average *z*-coherence of motor units within alpha band were correlated with improvements in performance during the skill acquisition task (i.e., RMSE between the force and target signals). Additionally, repeated-measures correlations were used to test whether changes in the average *z*-coherence of motor units within alpha band were associated with changes in the average *z*-coherence between force/neural drive and target template. These analyses were implemented using the *rmcorr* package with fixed slopes to estimate a single correlation coefficient for all participants. For this analysis, the data of TA and FDI muscles were pooled together. For all statistical comparisons, statistical significance was set at an *α* of 0.05. For the results of motor unit discharge properties, the values in the text are reported as mean with 95% confidence intervals. All the other values are reported as mean ± standard deviation in the text and median/interquartile ranges in the figures. All individual data of motor unit discharge times for both TA and FDI muscles recorded in the pre- and post-skill acquisition trials are available at https://doi.org/10.6084/m9.figshare.23703804. All effect sizes, along with their 95% confidence intervals and the methods of calculation, are detailed in the statistical table provided as supplementary material (Extended Data 1). Superscript lowercase letters in the Results section correspond to specific lines within this statistical table.

10.1523/ENEURO.0117-24.2024.d1 Extended Data 1 **Statistical table.** The statistical table provided contains all effect sizes, along with their 95% confidence intervals, and details regarding the methods of calculation. Download Extended Data 1, DOCX file.

### Code accessibility

The codes used for the computational model described in the paper are openly accessible online at https://doi.org/10.6084/m9.figshare.23703804. Additionally, these codes are provided in the supplementary material (Extended Data 2). The computations were performed on a Windows desktop with an AMD Ryzen 9 5950X 16-core processor and 128 GB of RAM.

10.1523/ENEURO.0117-24.2024.d2 Extended Data 2 **Computational model codes**. The codes utilized for the computational model described in the paper are provided here. The primary code is “*motoneuron_model_skill_training_paper.m*”. This file contains the main parameters of the simulation. Download Extended Data 2, ZIP file.

## Results

### Changes in force-matching and force power spectrum with skill acquisition

**Figure 3.**
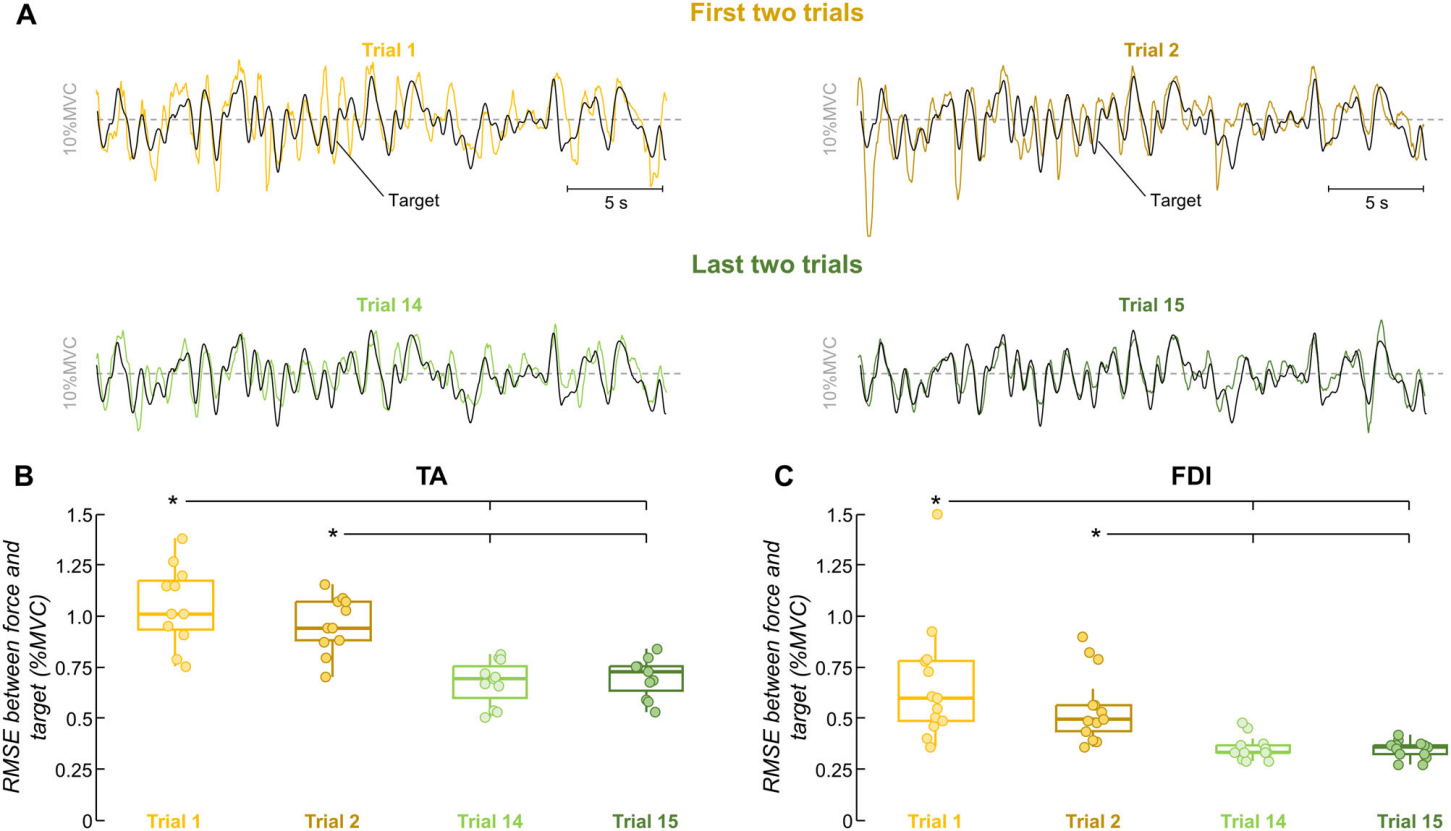
Performance results. Four (first two and last two) out of the 15 trials were used for each participant to assess improvements in force-matching. ***A***, Representative comparison between the force and target during the skill acquisition task. The yellow lines indicate dorsiflexion isometric forces produced by a participant for the first two trials, and the green lines for the last two trials. The black line indicates the target. ***B***, ***C***, Group results of RMSE between the force and target for the tibialis anterior (TA) muscle (***B***) and the first dorsal interosseous (FDI) muscle (***C***). Circles identify individual participants. Horizontal traces, boxes, and whiskers denote the median value, interquartile interval, and distribution range. *p < 0.05.

To assess improvements in force-matching during the skill acquisition task, we compared the RMSE and cross-correlation peak values between the force and target signals in the first two trials and the last two trials out of the 15. Figure 3*A* shows the fluctuations in dorsiflexion isometric force produced by a representative participant for these trials, where the yellow traces represent the first two trials, and the green traces refer to the last two. Improvements in force-matching are evident across trials, which are visually confirmed by the greater overlap between the force and the target template when comparing the final two with the initial two trials. Indeed, for both TA and FDI muscles, there were significant differences in RMSE (*p *< 0.001 for both muscles; Friedman test) and cross-correlation peak values (*p *< 0.001 for both muscles; Friedman test) among trials. Specifically, for both muscles, RMSE values were significantly lower in the last two trials compared with the first two (*p *< 0.006 for all; Bonferroni’s post hoc test; Fig. 3*B*,*C*)^a,b^. Moreover, for both muscles, cross-correlation peak values obtained in the last two trials were significantly greater than in the first two (*p *< 0.006 for all; Bonferroni’s post hoc tests)^c,d^. No significant differences were found in RMSE and cross-correlation within both the initial two trials and the final two trials (*p *> 0.128 for all; Bonferroni’s post hoc tests)^a–d^. Given this lack of difference and with the aim of maximizing the number of tracked motor units between trials (see Materials and Methods), for all subsequent analyses, we selected one trial from the first two (the one with the highest RMSE) and one trial from the last two (the one with the lowest RMSE) to represent the pre- and post-skill acquisition trials.

**Figure 4.**
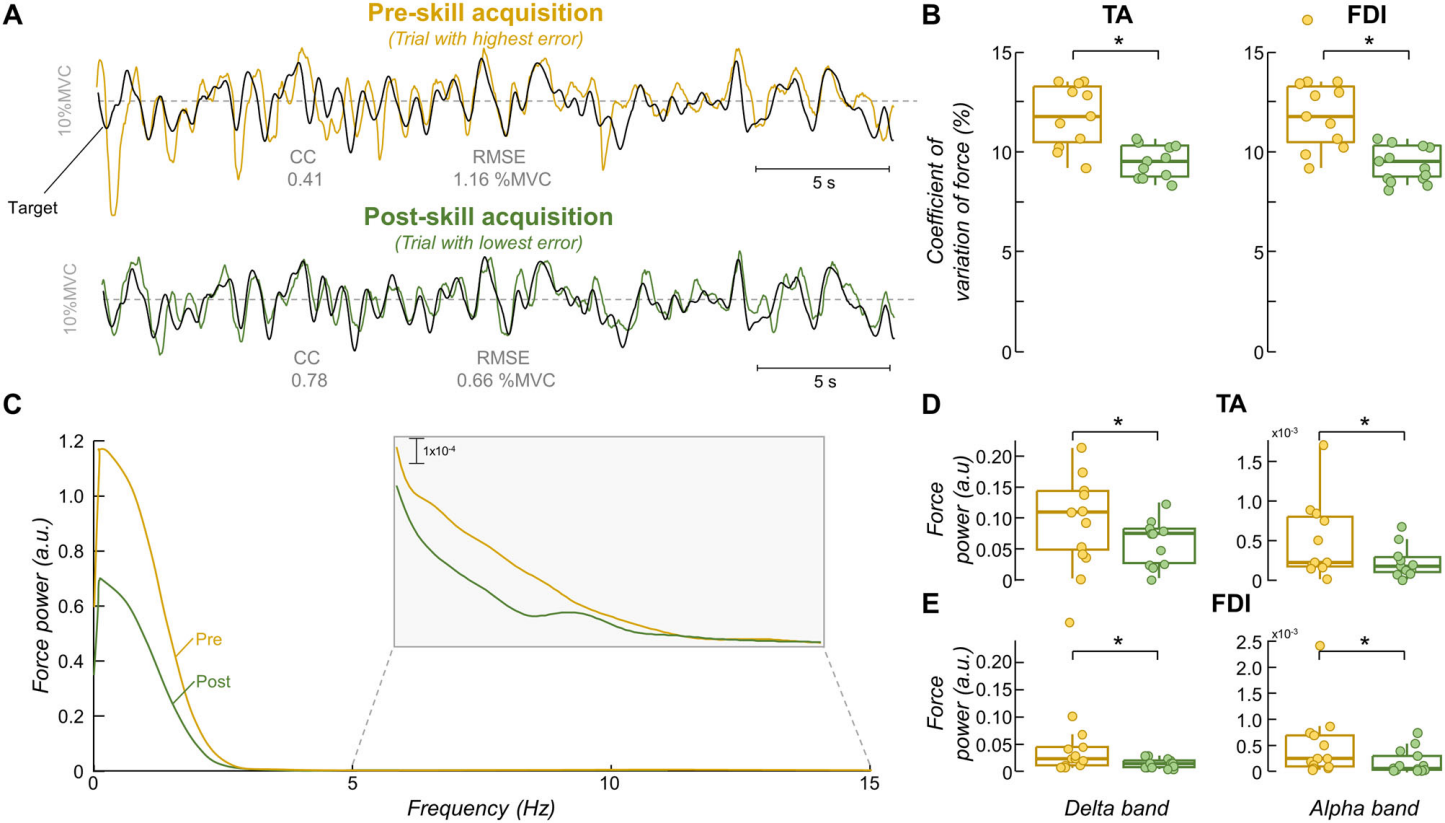
Force steadiness and force power spectrum results. Two trials were selected for each participant to represent the pre- and post-skill acquisition trials. ***A***, Representative comparison between the force and target for these two trials, where the yellow and green lines indicate the pre- and post-skill acquisition trials, respectively. The black line indicates the target. ***B***, Group results of coefficient of variation of force (force steadiness). ***C***, Power spectrum of force signals depicted in ***A***. The gray box shows a zoom in the alpha band (5–15 Hz). ***D***, ***E***, Group results of mean force power the tibialis anterior (TA) muscle (***D***) and the first dorsal interosseous (FDI) muscle (***E***). Circles identify individual participants. Horizontal traces, boxes, and whiskers denote median value, interquartile interval, and distribution range. *p < 0.05.

In Figure 4*A*, it is possible to see the two trials that were chosen to represent the pre- and post-skill acquisition trials for the same participant in Figure 3*A*. There is a decrease of ∼43% in RMSE (from 1.16 to 0.66%MVC) and an increase of ∼88% in cross-correlation peak value (from 0.41 to 0.78) between pre- and post-acquisition trials. To assess whether the force steadiness changed with the force-matching skill acquisition, we quantified the coefficient of variation of force for the two selected trials. For both TA and FDI muscles, there were significant differences in the coefficient of variation of force between trials (Fig. 4*B*). Specifically, the coefficient of variation of force values obtained for the post-skill acquisition trial were significantly lower than pre-skill acquisition trial for both TA (pre: 11.77 ± 1.57%; post: 9.51 ± 0.83%; *p *= 0.005; Wilcoxon signed-rank test)^e^ and FDI (pre: 14.13 ± 6.18%; post: 8.65 ± 0.93%; *p *< 0.001; Wilcoxon signed-rank test)^e^. We also examined how mean force power changed during the learning task, separately for the delta (1–5 Hz) and alpha (5–15 Hz) bands (the frequency bandwidth of the force signal). Figure 4*C* illustrates the power spectrum of force signals depicted in Figure 4*A*. It is visually evident for this representative participant that there was a reduction in the power spectrum of force between pre- and post-skill acquisition trials for both frequency bandwidths. This reduction in the mean force power with the force-matching skill acquisition was statistically confirmed for both TA (*p *< 0.003 for both delta and alpha bands; Wilcoxon signed-rank test; Fig. 4*D*)^f^ and FDI (*p *< 0.002 for both delta and alpha bands; Wilcoxon signed-rank test; Fig. 4*E*)^g^ muscles.

### Changes in motor unit discharge properties with skill acquisition

In order to evaluate changes in motor unit discharge properties (i.e., mean discharge rate and COV_ISI_) with the force-matching skill acquisition, we decomposed HDsEMG signals into motor unit spike trains. Note that we tracked motor units pre- and post-skill acquisition to ensure the same motor units were compared between trials (see the Materials and Methods section for further details). For the TA muscle, we identified a total of 166 and 198 motor units for pre- and post-skill acquisition trials, respectively, and were able to track 138 motor units (13 ± 7 motor units per participant). For the FDI muscle, instead, we identified a total of 105 and 127 motor units for pre- and post-skill acquisition trials, respectively, from which 62 were tracked across trials (5 ± 2 motor units per participant).

**Figure 5.**
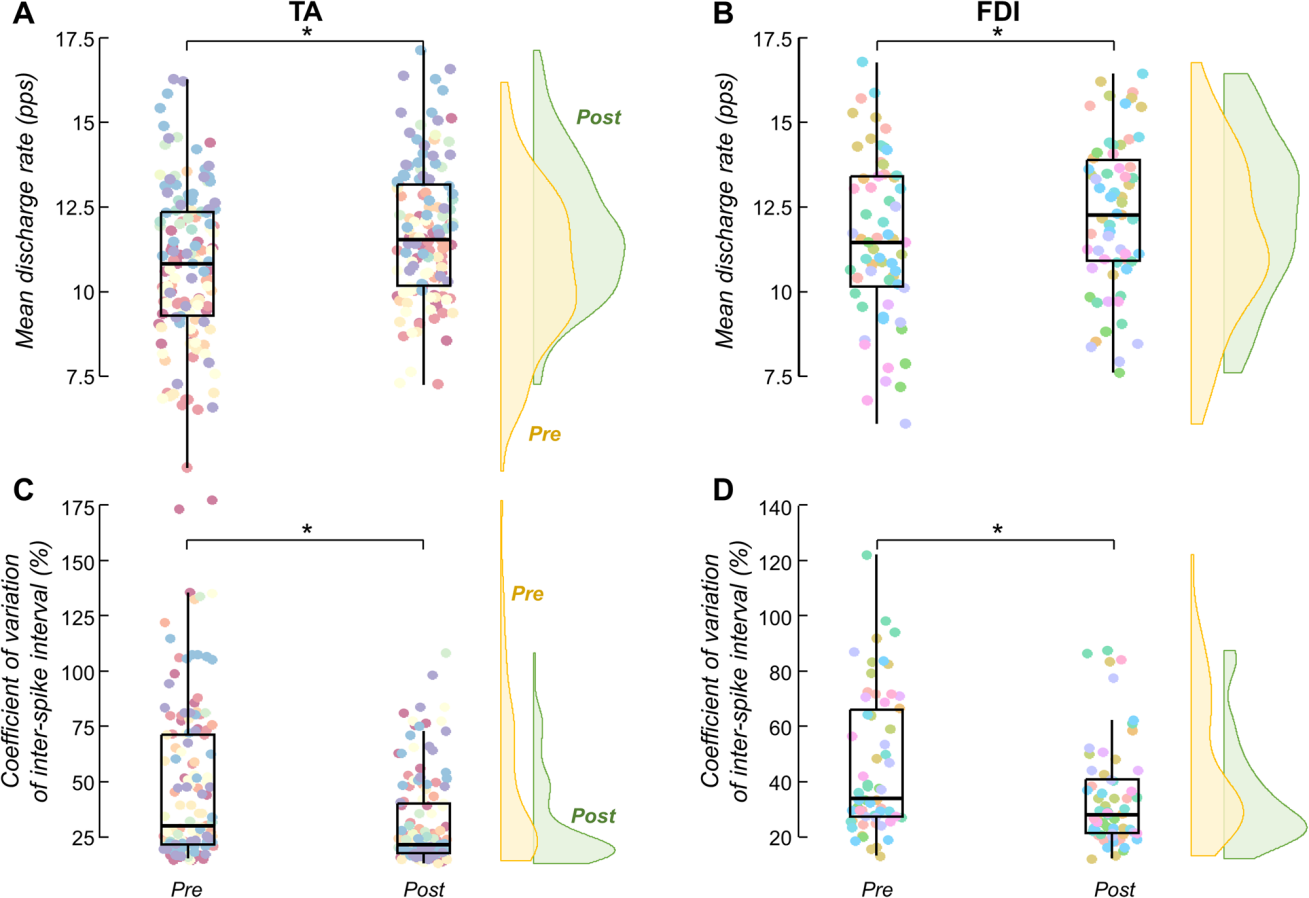
Mean discharge rate and discharge variability results. ***A***, ***B***, Mean discharge rate results of matched motor units between pre- and post-skill acquisition trials for the tibialis anterior (TA) muscle (***A***) and the first dorsal interosseous (FDI) muscle (***B***). ***C***, ***D***, Coefficient of variation of interspike interval results of matched motor units between pre- and post-skill acquisition trials for the TA (***C***) and FDI (***D***) muscles. Each circle identifies a matched motor unit between trials. Each color of the circles corresponds to a specific participant. Horizontal traces, boxes, and whiskers denote median value, interquartile interval, and distribution range. Density curves of the data are represented on the right side of each panel by half-violin plots (yellow for pre-skill acquisition and green for post-skill acquisition). Note that density curves can be used to visually compare differences between pre- and post-skill acquisition trials. *p < 0.05.

For both muscles, a significant effect of trial (pre- vs post-skill acquisition) was found on the mean discharge rate and COV_ISI_ values of matched motor units. Specifically, for the TA muscle, the mean discharge rate significantly increased from 10.9 [9.95, 11.9] to 11.9 [10.92, 12.9] pps between pre- and post-skill acquisition trials [*F *= 23.083; *p *< 0.001; linear mixed models (LMM); Fig. 5*A*]^h^. Conversely, the COV_ISI_ significantly decreased from 48.0 [42.9, 53.2] to 31.4 [26.3, 36.3]% with the force-matching skill acquisition (*F *= 22.986; *p *< 0.001; LMM; Fig. 5*C*)^i^. Similar results were observed for the FDI muscle, with the values of mean discharge rate significantly increasing from 11.6 [10.5, 12.7] to 12.3 [11.3, 13.4] pps (*F *= 5.166; *p *= 0.025; LMM; Fig. 5*B*)^j^ and the values of COV_ISI_ significantly decreasing from 46.6 [39.4, 53.8] to 35.4 [28.1, 42.6]% (*F *= 8.952; *p *= 0.003; LMM; Fig. 5*D*)^k^ between pre- and post-skill acquisition trials.

### Changes in common synaptic input with skill acquisition

**Figure 6.**
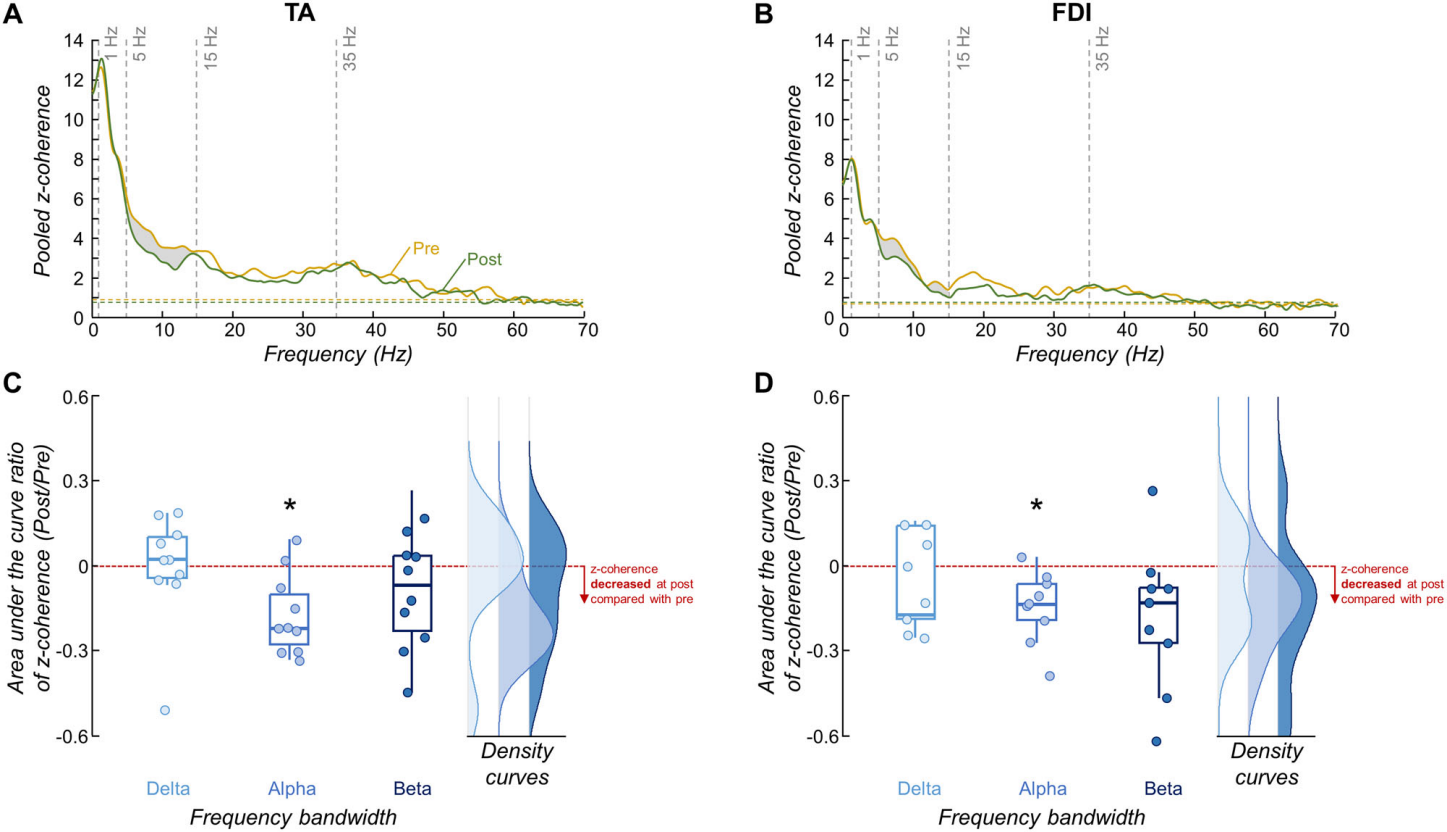
Motor unit coherence results. ***A***, ***B***, Pooled *z*-coherence profiles considering all participants for the tibialis anterior (TA) muscle (***A***) and first dorsal interosseous (FDI) muscle (***B***; yellow for pre-skill acquisition and green for post-skill acquisition). The horizontal dashed line indicates the confidence level. Vertical dashed lines highlight the three frequency bandwidths analyzed: delta (1–5 Hz), alpha (5–15 Hz), and beta (15–35 Hz) bands. Gray areas denote statistical differences in the area under the curve between pre- and post-skill acquisition trials. ***C***, ***D***, Group results of the area under the curve ratio of coherence for the TA (***C***) and FDI (***D***) muscles. Circles identify individual participants. Note that, for visualization purposes, the individual data point of one participant in the delta band of panel ***D*** (with a value exceeding 0.6) is not displayed. Horizontal traces, boxes, and whiskers denote median value, interquartile interval, and distribution range. Density curves of the data are represented on the right side of each panel by half-violin plots. *p < 0.05.

In order to assess changes in common synaptic input between pre- and post-skill acquisition trials, we used coherence analysis between the spike trains of matched motor units. We specifically quantified the ratio (post/pre) of the area under the curve of *z*-coherence within delta (1–5 Hz), alpha (5–15 Hz), and beta (15–35 Hz) bands. The coherence analysis was performed in 10 and 9 participants for TA and FDI muscles, respectively, as only three or fewer motor units were matched for Participant 7 in TA and Participants 2, 8, 11, and 13 in FDI. Figure 6, *A* and *B*, displays the pooled *z*-coherence for all participants. When comparing pre- and post-skill acquisition trials, for both TA and FDI muscles there is a clear reduction in the area under the curve in the alpha band (Fig. 6*A*,*B*, gray area). Indeed, for the TA muscle, there was a significant median reduction of ∼22% in the area under the curve within the alpha band between pre- and post-skill acquisition trials (*p *= 0.014; one-sample Wilcoxon signed-rank test; Fig. 6*C*)^l^, which was not observed either for delta or beta bands (*p *> 0.322 for both; one-sample Wilcoxon signed-rank tests)^l^. Similarly, for the FDI muscle, the area under the curve within the alpha band significantly decreased by a median of ∼13% with the force-matching skill acquisition (*p *= 0.008; one-sample Wilcoxon signed-rank test; Fig. 6*D*)^m^ but did not significantly change for delta or beta bands (*p *> 0.074 for both; one-sample Wilcoxon signed-rank tests)^m^. Notably in Figure 6*D*, two potential outlier values are visible in the beta band (dots higher than 0 and close to −6). When excluding these values from the analysis, there was a significant decrease in the area under the curve within beta band between pre- and post-skill acquisition trials for the FDI muscle (*p *= 0.02; one-sample Wilcoxon signed-rank test).

To confirm that the observed reductions in alpha band coherence with the force-matching skill acquisition were not solely attributable to motor execution independent of learning, we collected data from a subgroup of three participants who repeated 15 trials of steady isometric contractions of dorsiflexion. In contrast to the changes observed between pre- and post-skill acquisition trials, there was an average increase of 56.1% in alpha band coherence during the repetition of motor execution, with individual increases of 107.3, 11.1, and 49.8%. Furthermore, to examine whether the absence of statistical differences within the beta band was due to the number of motor units utilized in the CST, we conducted an additional analysis using the TA motor unit data (for detailed information, please refer to the Materials and Methods section). In brief, we calculated *z*-coherence between two CSTs, incrementally increasing the number of motor units in each CST from 1 to 13 (half of the maximal number of matched motor units across all participants). We then computed the area under the curve ratio (post/pre) of *z*-coherence within the beta band. If our results within the beta band were influenced by the number of motor units used in the CSTs, we would expect to observe a significant deviation from zero in this ratio curve as the number of motor units increased. Instead, we observed that the results of area under the curve ratio of *z*-coherence within the beta band were not influenced by the number of motor units used in the CSTs, which was confirmed statistically using the one-sample Statistical Parametric Mapping (*p *> 0.05).

### Changes in coherence between force/neural drive and target with skill acquisition

**Figure 7.**
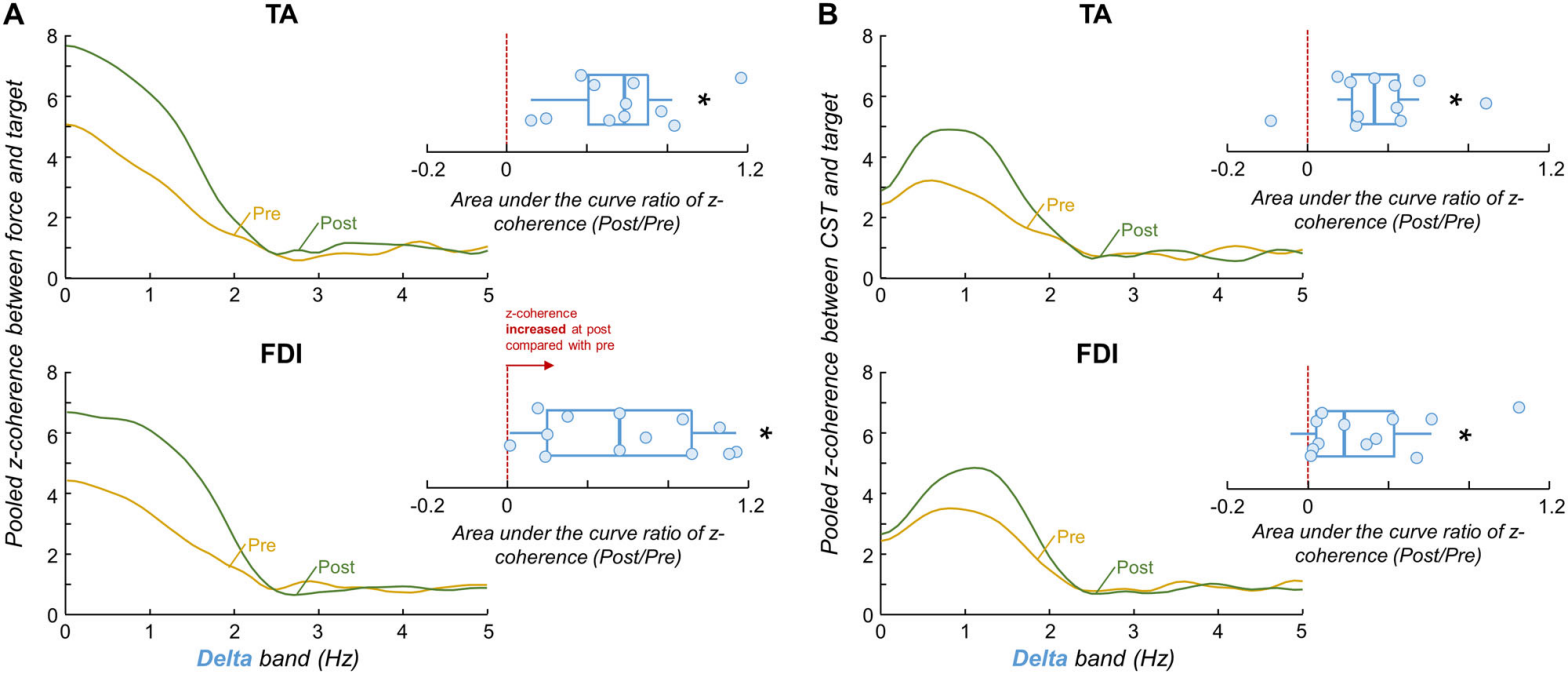
Coherence between force/neural drive and target results. Pooled *z*-coherence profiles between force and target (***A***) and CST and target (***B***) for the tibialis anterior (TA) muscle (top) and first dorsal interosseous (FDI) muscle (bottom). Yellow and green lines indicate the pre-skill acquisition and post-skill acquisition trials, respectively. Note that the frequency bandwidth analyzed was only the delta band (the frequency bandwidth of the target). Blue boxplots show the group results of the area under the curve ratio of *z*-coherence. Circles identify individual participants. Horizontal traces, boxes, and whiskers denote median value, interquartile interval, and distribution range. *p < 0.05.

To evaluate changes in the linear coupling between oscillations in force/neural drive and oscillations in the target template between pre- and post-skill acquisition trials, we calculated the *z*-coherence within delta band (the frequency bandwidth of the target) between the target and the force and between the target and the neural drive to the muscles (i.e., CST). Figure 7 displays the pooled *z*-coherence between force and target, and between CST and target, for both TA (top) and FDI (bottom) muscles. In all cases, there was a clear increase in the area under the curve of *z*-coherence between pre- and post-skill acquisition trials. Indeed, significant increases in the area under the curve ratio of target–force *z*-coherence (*p *< 0.001 for both muscles; one-sample Wilcoxon rank-signed test; Fig. 7*A*)^n^ and target–CST *z*-coherence (*p *< 0.004 for both muscles; one-sample Wilcoxon rank-signed test; Fig.7*B*)^o^ were observed between pre- and post-skill acquisition trials.

**Figure 8.**
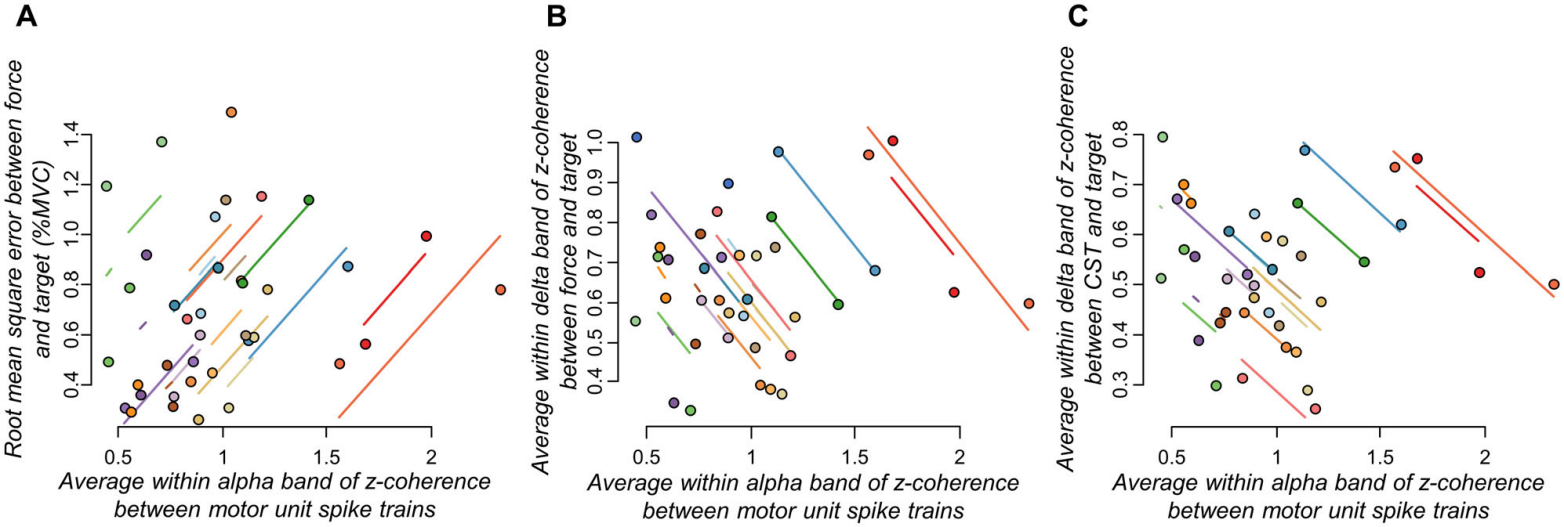
Correlation results. Repeated-measures correlations between changes in motor unit coherence within the alpha band and root mean square error between force and target signals (***A***), as well as between motor unit coherence within alpha band and coherence between force and target (***B***), and between motor unit coherence within the alpha band and coherence between CST and target (***C***). For this analysis, data from tibialis anterior and first dorsal interosseous muscles were pooled together.

To investigate whether reductions in the motor unit *z*-coherence within the alpha band between trials (Fig. 6) were correlated with improvements in performance (Fig. 3), as well as changes in target–force and target–CST coherence between trials (Fig. 7), we used repeated-measures correlations. For this analysis, the data from TA and FDI muscles were pooled together. Figure 8*A* shows a significant association between average *z*-coherence within alpha band and RMSE between force and target signals (*r*_rm_ = 0.574; *p *= 0.008)^p^. Furthermore, significant inverse associations were observed between average *z*-coherence within alpha band and target–force average *z*-coherence (Fig. 8*B*; *r*_rm_ = −0.638; *p *= 0.002)^q^, as well as between average *z*-coherence within alpha band and target–CST average *z*-coherence (Fig. 8*C*; *r*_rm_ = −0.619; *p *= 0.004)^r^.

### Neural mechanisms underlying the short-term acquisition of a skill task

**Figure 9.**
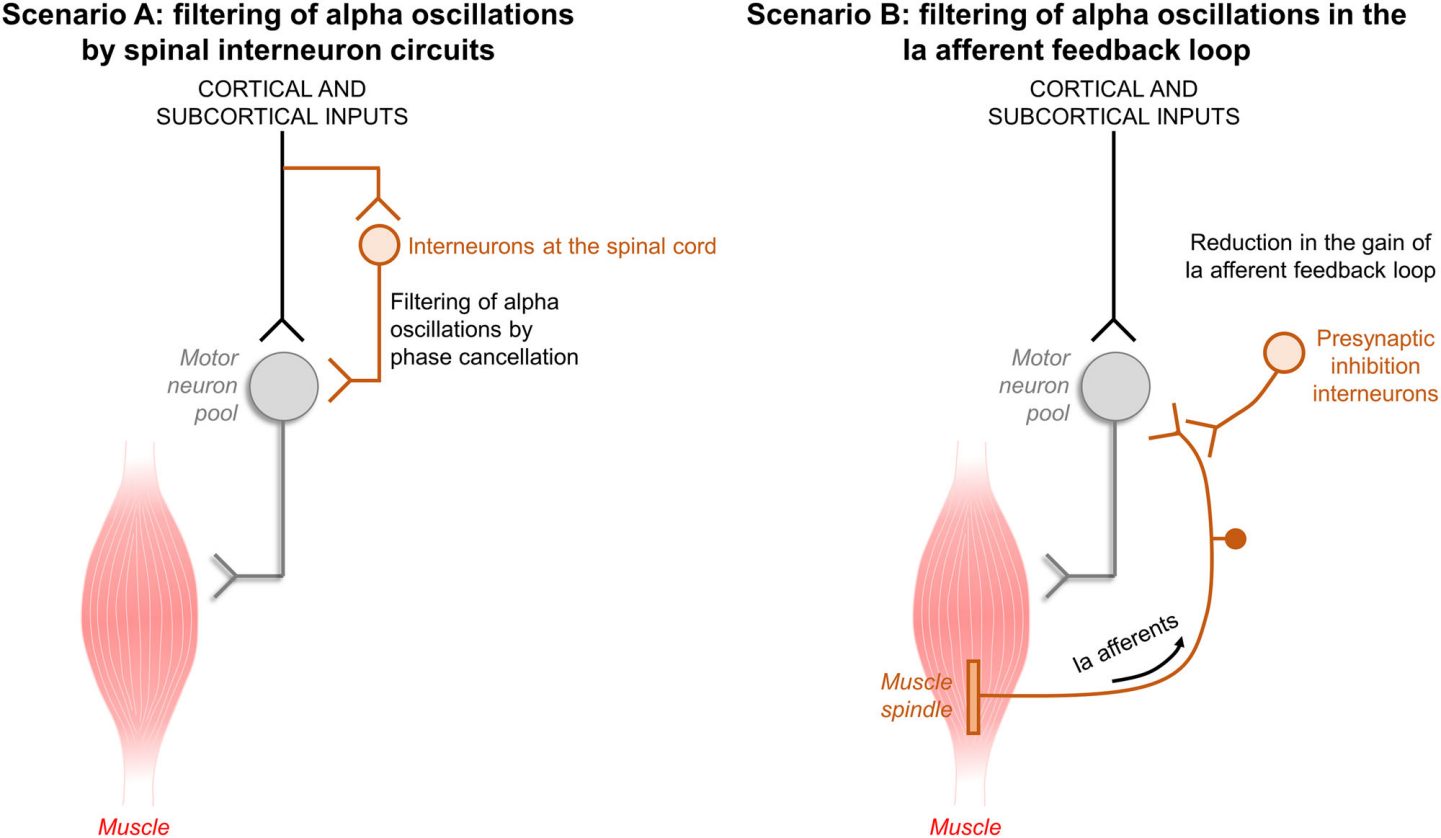
Simulated scenarios to investigate neural mechanisms underlying the experimental results. Two different scenarios were simulated to investigate potential mechanisms that could explain the observed changes between pre- and post-skill acquisition. In Scenario ***A*** (left panel), we hypothesized that decreases in alpha band with the acquisition of the force-matching skill could be explained by spinal interneurons phase-cancelling central oscillatory inputs in the alpha frequency range. In Scenario ***B*** (right panel), we hypothesized that reductions in alpha band with the force-matching skill acquisition could be explained by increases in presynaptic inhibition of Ia afferent feedback into the motor neuron pool. Details about how we simulated these scenarios are provided in the Materials and Methods section.

To explore the neural mechanisms underlying the observed changes between pre- and post-skill acquisition, we simulated a population of motor neurons receiving common and independent inputs and the sequence of events from the excitation of these motor neurons to the generation of isometric force output. Specifically, two different scenarios were simulated (refer to Materials and Methods section for further details). In Scenario A, we hypothesized that decreases in alpha band with the acquisition of the force-matching skill could be explained by spinal interneurons phase-cancelling central oscillatory inputs in the alpha frequency range (Fig. 9*A*). In Scenario B, the reductions in alpha band with the force-matching skill acquisition could be explained by increases in presynaptic inhibition of Ia afferent feedback into the motor neuron pool (Fig. 9*B*). We calculated the simulated force output power spectrum; the ratio (post/pre) of the area under the curve of motor unit *z*-coherence within delta (1–5 Hz), alpha (5–15 Hz), and beta (15–35 Hz) bands; and the area under the curve ratio (post/pre) of *z*-coherence between simulated force/CST and the target template. We then compared the results of the different scenarios with the experimental results to explore which simulated scenario aligns more closely with the observed experimental outcomes.

**Figure 10.**
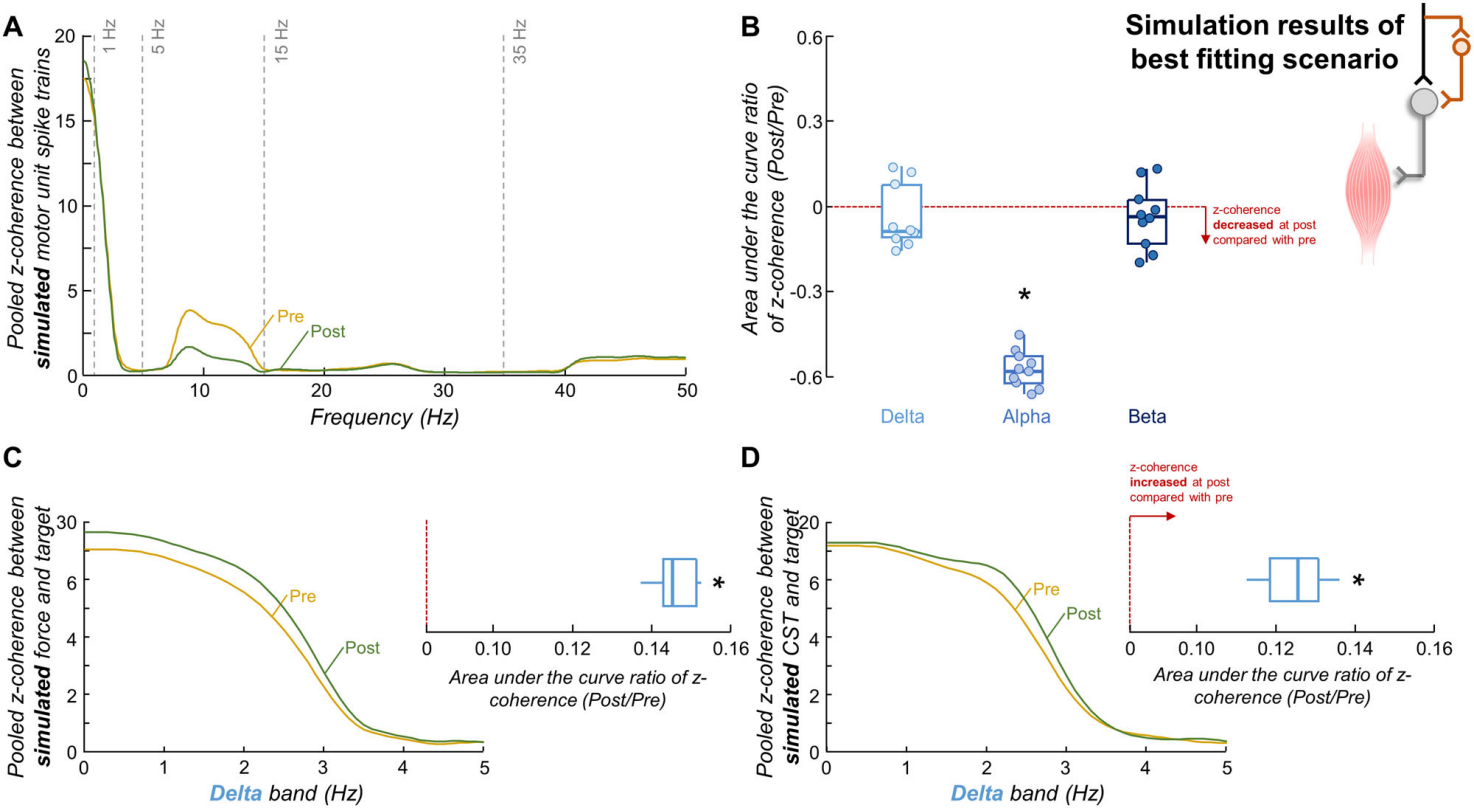
Simulation results of best fitting scenario. ***A***, Pooled *z*-coherence profiles considering all realizations. Vertical dashed lines highlight the three frequency bandwidths analyzed: delta (1–5 Hz), alpha (5–15 Hz), and beta (15–35 Hz) bands. ***B***, Group results of the area under the curve ratio of coherence. Circles identify individual simulation realizations. Horizontal traces, boxes, and whiskers denote median value, interquartile interval, and distribution range. ***C***, ***D***, Pooled *z*-coherence profiles between simulated force and target (***C***) and between simulated CST and target (***D***). Note that the frequency bandwidth analyzed was only the delta band (the frequency bandwidth of the target). Blue boxplots show the results of the area under the curve ratio of *z*-coherence for all simulation realizations. In panels ***A***, ***C***, and ***D***, yellow and green lines indicate the pre-skill acquisition and post-skill acquisition models, respectively. *p < 0.05.

Results of Scenario A were found to align more closely with the observed experimental outcomes, but only when we slightly increased the beta band input to the motor neuron pool in the post-skill acquisition model compared with pre-skill acquisition model. Consistent with the experimental results, there was a reduction in the power spectrum of force between pre- and post-skill acquisition models for both delta and alpha bands (*p* < 0.05 for both; Wilcoxon signed-rank test). Figure 10*A* displays the pooled *z*-coherence of simulated motor units for the 10 realizations of the best fitting scenario (Scenario A), showing a clear reduction within alpha band between pre- (yellow line) and post-skill acquisition (green line) models. Indeed, the area under the curve of coherence within the alpha band significantly decreased between pre- and post-skill acquisition models (*p *< 0.002; one-sample Wilcoxon signed-rank test; Fig. 10*B*) but did not significantly change either for delta or beta bands (*p *> 0.274 for both; one-sample Wilcoxon signed-rank tests; Fig. 10*B*). Moreover, there were an increase in the area under the curve of simulated target–force *z*-coherence (*p *= 0.002; one-sample Wilcoxon rank-signed test; Fig. 10*C*) and simulated target–CST *z*-coherence (*p *= 0.002; one-sample Wilcoxon rank-signed test; Fig. 10*D*) between pre- and post-skill acquisition models. In contrast, the simulation results of Scenario B did not match the experimental results. In this scenario, there was a significant increase in the power spectrum of force within delta band (*p *= 0.002; Wilcoxon signed-rank test), as well as a significant increase in the area under the curve of coherence within the delta band (*p *= 0.002; one-sample Wilcoxon signed-rank test) between pre- and post-skill acquisition models, which were not observed in the experimental outcomes.

## Discussion

In this study, we investigated whether short-term learning of a complex, isometric force-matching task is mediated by specific adaptations in the shared synaptic inputs to alpha motor neuron pools of the TA and FDI muscles. Our experimental findings revealed that both muscles exhibited improvements in force-matching as skill acquisition progressed, accompanied by a reduction in coherent oscillations across motor neuron spike trains unrelated to the required force fluctuations (i.e., physiological tremor band oscillations). Importantly, these reductions in alpha band with the force-matching skill acquisition correlated significantly with improvements in performance and an increased coupling between force/neural drive and target oscillations. Based on simulations, our findings further indicate that the potential neural mechanism underlying decreases in alpha band with the acquisition of the force-matching skill is related to spinal interneurons phase-cancelling central oscillatory inputs in the alpha frequency range. As discussed below, these outcomes suggest that the acquisition of a new force-matching task involves specific changes in spinal neural circuitry that behave as a matched neural filter, ultimately minimizing shared noise components unrelated to the intended task.

Traditionally, motor sequence learning, which assesses the incremental acquisition of sequential motor skills, has been used as experimental paradigm to investigate neuroplasticity underlying skilled task acquisition (Karni et al., 1998; Ungerleider et al., 2002). Psychophysiological evidence has revealed that two main stages are involved in this paradigm: a fast stage characterized by considerable performance improvements within a single session and a slow stage where further but quantitatively smaller improvements can be observed across multiple sessions (Ungerleider et al., 2002; Dayan and Cohen, 2011). Consistent with previous research (Knight and Kamen, 2004; Perez et al., 2005), our study focused on the fast-learning stage of incremental motor skill acquisition, as we aimed to investigate the rapid acquisition of a fine control task involving muscle force modulation. Thus, ensuring that the observed neural plastic changes in this study were specifically related to the force-matching skill task, and did not arise from overall motor activity or other confounding factors, is a necessary step for interpreting our main results. The median reductions in RMSE of ∼40% for both the TA and FDI observed after 15 repetitions of the task (Fig. 3), along with cross-correlation increases of ∼35% for both muscles, indicate that the chosen task and number of trials were sufficiently challenging to enable skill acquisition. Previous experiments using a similar task involving index finger abduction demonstrated comparable RMSE improvements (∼50%) between force and target over the same number of repetitions (Knight and Kamen, 2004). Taking these factors into consideration, it is plausible to assume that the alterations identified in motor unit discharge characteristics and shared synaptic inputs are indeed associated with the intended force-matching task.

Due to the low-pass and amplification characteristics of the motor neuron pool, experimental and simulated data have extensively demonstrated that only the low-frequency components of the synaptic inputs largely shared across the motor neuron pool are represented in the generated muscle force (Negro et al., 2009, 2016b; Negro and Farina, 2011a,b, Farina et al., 2014; Thompson et al., 2018). Although the contributions of these common synaptic input components may not be directly measured in humans, they have been indirectly estimated through coherence analysis of discharge times of motor unit spike trains (Negro and Farina, 2012; Castronovo et al., 2015; Maillet et al., 2022; Rossato et al., 2022). For instance, Castronovo et al. (2015) have demonstrated significant coherence between motor unit spike trains up to ∼80 Hz during isometric contractions, and these results have been repeatedly confirmed by subsequent studies using EMG (Kerkman et al., 2018) and motor unit (McManus et al., 2019; Muceli et al., 2022) recordings. Among these significant oscillation frequencies, the delta band of coherence (1–5 Hz) is believed to reflect the effective control signal to the motor neuron pool (i.e., task-related oscillations of the common synaptic input) as it encompasses the frequency range of the force signal (Farina et al., 2014). On the other hand, beta band oscillations (15–35 Hz) in motor unit coherence are commonly attributed to cortical origin, as studies have demonstrated associations between muscular and cortical activities within this frequency range (Conway et al., 1995b; Baker et al., 1997; Baker, 2007). Finally, the alpha band coherence (5–15 Hz) is typically associated with the physiological tremor (Conway et al., 1995a; Christakos et al., 2006; Laine et al., 2016).

Given that alpha band frequencies are not entirely filtered out by the contractile properties of the muscle (Bawa and Stein, 1976; Baldissera et al., 1998), they are also reflected in the variability of force. Thus, these neural oscillations can be viewed as an involuntary common noise input that limits the accuracy of the force output (i.e., task-unrelated oscillations of the common synaptic input). For instance, a demanding visuomotor task has been shown to increase alpha band coherence, which was accompanied by larger force tremors (Laine et al., 2014). These findings, along with others (Ribot-Ciscar et al., 2009; Roche et al., 2011), suggest that a high-sensitivity task, such as the one presented to the participants in this study, would result in larger fluctuations in the alpha band and consequently, lead to increased force oscillations. Indeed, as illustrated in Figure 4*A* and reflected in the coefficient of variation of force results (Fig. 4*B*), the pre-skill acquisition trial exhibited greater force fluctuations in both the TA and FDI muscles. Supporting this observation, we also found higher motor unit discharge variability, a measure of the variance of the common synaptic input received by the motor neurons, in the pre-skill acquisition trial (Fig. 5*C*,*D*), which is consistent with previous research (Knight and Kamen, 2004; Ely et al., 2022). However, we hypothesized that the repetition of this challenging task would prompt the central nervous system to effectively minimize the common synaptic oscillations unrelated to the specific task. This would lead to reductions in alpha band oscillations (physiological tremor), subsequent improvements in force control, and, ultimately, the short-term acquisition of the motor task.

To test our hypothesis, we experimentally examined changes in motor unit coherence within delta, alpha, and beta bands between pre- and post-skill acquisition. For both investigated muscles, our results revealed reductions in *z*-coherence, specifically within the physiological tremor frequency band (Fig. 6), supporting our hypothesis. Importantly, these reductions were reflected in the oscillations of muscle force output within the alpha band (Fig. 4*C–E*). To further investigate whether these decreases in physiological tremor frequencies in both neural and force outputs were indeed associated with the force-matching skill acquisition, we calculated the linear coupling between the target and the force, as well as between the target and the CST (an estimation of the effective neural drive to the muscle). We found that both the target–force and target–CST coherence values increased significantly between pre- and post-skill acquisition (Fig. 7), indicating an enhancement in the representation of task-related oscillations of shared synaptic input with the force-matching skill acquisition. Notably, this better match between the neural/mechanical oscillations and the target fluctuations correlated significantly with the observed reductions in alpha band (physiological tremor) oscillations (Fig. 8*B*,*C*). These findings collectively support our hypothesis that, indeed, the short-term acquisition of a force-matching skill is mediated by a reduction of physiological tremor in motor neuron inputs.

The observed decrease in alpha band oscillations could be attributed to modulations in peripheral pathways, cortical pathways, or both. The possibility of peripheral modulation aligns with previous studies showing H-reflex depression following visuomotor skill tasks (Perez et al., 2005; Giboin et al., 2020), suggesting an increase in the presynaptic inhibition of Ia afferents during motor skill acquisition. Considering the involvement of Ia afferent loop in physiological tremor enhancement (Cresswell and Löscher, 2000; Christakos et al., 2006; Laine et al., 2016), this increased Ia inhibition with skill acquisition may underlie the observed reductions in physiological tremor oscillations (Fig. 9, Scenario B). Another possibility is that these decreases in physiological tremor band occur via central pathways. This possibility is consistent with previous evidence showing that spinal interneurons could reduce alpha band oscillations in motor neuron output by phase-inverting the inputs to motor neurons at this frequency bandwidth (Williams et al., 2010; Koželj and Baker, 2014). These spinal interneurons would act as a neural filter, cancelling the descending oscillations within alpha band. Therefore, it is possible that the gain of this spinal interneuron filter undergoes upregulation during force-matching skill acquisition, consequently amplifying the cancellation effect (Fig. 9, Scenario A). To explore which of these two possibilities aligns more closely with our experimental outcomes, we conducted simulations. Our findings demonstrated that the spinal interneurons filter, cancelling cortical oscillatory inputs in the alpha frequency range, is likely the explanation for our experimental findings, as all the results of this simulation scenario were similar to the experimental observations (Fig. 10). Conversely, reductions in the gain of Ia afferent feedback loop alone are unlikely to explain the observed reductions in alpha band oscillations with the acquisition of the force-matching skill.

It is still possible that the observed decreases in alpha band oscillations are mediated by direct alterations in cortical oscillatory activity during the acquisition of the force-matching skill. Although supraspinal projections to alpha motor neurons originate from various sources (cortical and subcortical pathways; Fig. 9), shared inputs from the motor cortex are believed to be the primary source of correlation between motor neuron spike trains during voluntary tasks (Conway et al., 1995b; Baker et al., 1997). According to the alpha inhibition hypothesis (Klimesch, 1996; Pfurtscheller, 2003), cortical alpha oscillations are presumed to play an active role in top-down inhibitory control of neuronal processes and in modulating cognitive functions such as perceptual learning [for a review, see Sigala et al. (2014)]. For instance, compelling evidence suggests that sensory tasks and self-paced movements may lead to diminished encephalography activity within alpha bands (Neuper and Pfurtscheller, 2001). Consequently, the changes in alpha band coherence observed in the present study at the neuromuscular level may reflect, at least partially, ongoing modulations in electrocortical brain activity transmitted to the motor neuron pools during the learning process of the visuomotor task. However, it is noteworthy that in healthy individuals, corticomuscular coherence does not show direct significant coherent oscillations in the alpha band (Conway et al., 1995b; Baker et al., 1997), and no changes in corticomuscular coherence in the alpha band were observed following a visuomotor training task (Perez et al., 2006). Therefore, we believe that alterations in cortical activity alone may not fully account for the decreased alpha band oscillations at the motor neuron pool level observed with skill learning. It is also plausible that more complex neural schemes of physiological tremor oscillation cancellation occur, combining cortical alterations and the two scenarios outlined in Figure 9 into a unified system or, for example, involving recurrent inhibition from spinal Renshaw cells, which could also remove the alpha band components of motor neurons output (Williams and Baker, 2009).

A final consideration of the shared synaptic alterations during the learning of a force-matching task is related to the alterations in beta band oscillations. Our experimental results showed no significant changes in beta band coherence between pre- and post-skill acquisition trials for both FDI and TA muscles (Fig. 6). Importantly, this lack of change was not attributed to a low number of tracked motor units between trials (see Results). However, in the simulation that best aligned with the experimental outcomes (Fig. 9, Scenario A), we could only replicate the experimental findings by simulating a slight increase in beta band oscillations in the shared synaptic inputs to motor neurons between pre- and post-skill acquisition models. Additionally, upon removing two potential outliers in the beta band (Fig. 6*D*), a weak but still significant reduction in beta band oscillations was observed with the force-matching skill for the FDI muscle. These results suggest that beta oscillations might still play a role in short-term acquisition of a force-matching skill task, consistent with recent evidence suggesting that cortical beta oscillations are associated, at least in part, with motor performance following visuomotor learning (Espenhahn et al., 2019). Further investigation is needed to elucidate the implications of beta oscillations in the context of a force-matching skill acquisition.

Finally, we observed a subtle increase in mean discharge rate (∼1 pps; Fig. 5*A*,*B*) with the force-matching skill acquisition. An increase in mean discharge rate has been linked to improved transmission of common synaptic input to motor neuron spike trains (Negro and Farina, 2012), which would result in increased shared synaptic oscillations. Contrarily, we observed a decrease in shared synaptic noise oscillations despite the increase in mean discharge rate. These results suggest that the observed increases in mean discharge rate are unlikely to explain the reductions in shared synaptic noise oscillations observed with force-matching skill acquisition. Hence, it is possible that changes in peripheral motor unit properties, such as reductions in motor unit twitch duration or amplitude, may have contributed to the observed changes in mean discharge rate. Another plausible possibility is the cocontraction of antagonistic muscles to stiffen the joints in response to the challenges of the learning task, inducing an increase in the mean discharge rate. Indeed, prior investigation has demonstrated that antagonistic cocontraction can enhance learning of novel motor tasks (Heald et al., 2018). However, further investigation is warranted to fully elucidate the underlying mechanisms.

From a practical perspective, our findings suggest that a brief (∼25 min) session of a force-matching task involving the tracking of low-frequency oscillations led to improvements in muscle force control and accuracy. These improvements in performance, evidenced by reductions in the coefficient of variation of force (Fig. 4*B*) and decreased error between force and target signals (Fig. 3*B*,*C*), were correlated with the observed decreases in alpha band oscillations in motor neuron inputs (Fig. 8*A*). Importantly, these reductions in the alpha band were not solely attributable to motor execution, as the control group exhibited opposite changes with increases in alpha oscillations during repeated motor execution independent of learning. This implies that the reduction in alpha band activity may represent one of the mechanisms underlying performance improvements during a force-matching skill learning task. Furthermore, considering we observed increases in alpha band activity after the repetition of an isometric task without learning, it is conceivable that the reductions in alpha band during the acquisition of a force-matching skill are even more significant. Therefore, the low-intensity task provided to healthy individuals in our study may hold relevance for clinical populations with pathological tremor, such as Parkinson's disease, and older adults, who often exhibited enhanced physiological tremor oscillations during force production (Semmler et al., 2003). Further investigations are warranted to explore the potential impact of the proposed force-matching task on these specific populations. Additionally, future research should explore the translational implications of our findings for long-term training scenarios. For instance, a 4 week training period involving a similar force-matching task (following a sinusoidal target) resulted in comparable improvements in performance (Ely et al., 2022), suggesting promising possibilities for further investigation.

In conclusion, our study demonstrates that the acquisition of a force-matching skill involves specific adaptations in the shared synaptic input to alpha motor neurons, leading to improved force control by minimizing physiological tremor oscillations in motor neuron inputs. Furthermore, our findings propose that the likely mechanism driving these reductions in alpha band oscillations involves spinal interneurons phase-cancelling the descending oscillations within alpha band. Therefore, our study provides novel insights into the neural mechanisms underpinning short-term motor learning.

## Synthesis

Reviewing Editor: David Franklin, Technische Universitat Munchen Decisions are customarily a result of the Reviewing Editor and the peer reviewers coming together and discussing their recommendations until a consensus is reached. When revisions are invited, a fact-based synthesis statement explaining their decision and outlining what is needed to prepare a revision will be listed below. The following reviewer(s) agreed to reveal their identity: Firas Mawase, Ronan Mooney.

Your manuscript has been reviewed by two experts in the field. They both appreciate the importance and timeliness of your work, which uses a force matching task and high-density surface EMG to investigate adaptations of spinal and supraspinal pathways. They particularly highlight the combination of experimental and modelling approaches to studying motor skill adaptation. However, we all have some concerns regarding the current version of the manuscript, particularly relating to the relation between the coherence of alpha reduction and performance improvement, the lack of a non-adaptation behavioural control condition, questions about cortical and subcortical contributions. Nevertheless, we would be very interested to consider a revised version of the manuscript. Please take into account these concerns, and the detailed feedback from the reviewers below, as you revise your manuscript.

General Comments from the Reviewers.

Reviewer 1:

This manuscript presents an interesting and timely exploration into the neural mechanisms underlying the acquisition of a new force-matching task, employing high-density surface electromyography (HDsEMG) to investigate the adaptations of spinal and supraspinal pathways. The used approach, combining experimental and modeling techniques, offers valuable insights into the synaptic contributions to motor skill learning, particularly through the activity of motor units (Mus) and neural oscillation patterns. The observed reduction in the alpha band and its correlation with performance improvement is compelling, highlighting a critical aspect of neural adaptation during motor learning. The models tested are plausible and lend substantial support to the main conclusions, indicating a thoughtful approach to validating the theoretical framework of the study. Overall, this is an interesting work with well-conducted experiments and clear results. However, there are several shortcomings that should be addressed in detail.

Major comments

The relationship between the coherence of alpha reduction and performance improvement is somewhat ambiguous and weakly presented. This core aspect of the study would benefit from further elucidation to solidify the arguments of the authors.

The assumption within the model that inputs from subcortical and cortical sources are equivalently processed is a significant oversimplification. The predominance of cortical inputs in such tasks should be more accurately reflected in the model to enhance its realism and applicability. At least discussing this important point would be beneficial.

Minor comments:

The Methods section, while detailed, might benefit from streamlining and the inclusion of visual aids to improve clarity and readability. Specifically, visual examples of motor unit spike train analysis could enhance comprehension.

Certain terms, such as "COV_ISI" and "CST," lack explicit definitions, which could pose a barrier to understanding for readers unfamiliar with the terminology.

The analysis in Figure 5D concerning the beta band's role warrants further investigation, particularly regarding the potential influence of outliers on the results. Clarification on this point could reinforce the study findings or reveal additional nuances in the data.

Additionally, it is recommended to reconsider the terminology used in the manuscript, particularly the use of "tremor" in the title and main conclusions. While the study's findings regarding the alpha band's involvement in motor skill acquisition are robust and significant, the term "tremor" may inadvertently suggest a focus on pathological conditions, potentially misleading readers about the participant population and the study's scope. Given that both alpha oscillations and tremor phenomena occupy similar frequency ranges and could be linked to similar neural processes, the term might be appropriate in a clinical or pathological context.

Reviewer 2:

The study assessed how shared synaptic input to spinal motor neurons are modulated during the acquisition of a novel motor skill. The authors found that the participants learned the task as exhibited by reduced error, force variability, and force power (in the delta and alpha band) from the start to the end of training. These behavioral changes were accompanied by increased motor unit discharge rate, decreased inter-spike interval variability, and decreased area under the curve within alpha band oscillations. The study is interesting, and the authors should be commended for their rigorous methodology and clear presentation of the data. Although, I have a few comments which should be considered.

Major points

1. A limitation of the present study is that the authors did not include a separate behavioral condition which controls for motor execution independent of learning. The authors state in the introduction that adaptations at the supraspinal level, such as increased corticospinal excitability, accompany motor skill acquisition. However, increases in corticospinal excitability can also be observed due to motor execution (see Spampinato &Celnik 2017 Sci Rep). It is possible that similar adaptation at the spinal level may also be observed following an equivalent period of motor execution independent of learning. This should be noted in the discussion.

2. How do these adaptations observed in the EMG align with supraspinal observations? There is some discussion about cortical oscillatory activity. However, the authors could expand more regarding how changes at the supraspinal level might be mediating these peripheral adaptations and vice-versa. For example, is increased corticospinal excitability beneficial if the "goal" of the system is to reduce unwanted noise (i.e., physiological tremors)?

3. How might these findings inform rehabilitation of clinical populations where behavioral phenotypes can impede performing everyday motor skills? Should these adaptations at the spinal level be assessed alongside supraspinal adaptations? Are there specific clinical populations in which these properties (e.g., physiological tremors) are abnormal and could be contributing to motor impairments? These points should be briefly considered in the discussion.
