# Supplementary material for: Neural Filtering of Physiological Tremor Oscillations to Spinal Motor Neurons Mediates Short-Term Acquisition of a Skill Learning Task
Source: eNeuro. 2024 Jul 17;11(7):ENEURO.0043-24.2024. doi: 10.1523/ENEURO.0043-24.2024 (PMC11255391; doi:10.1523/ENEURO.0043-24.2024)
Supplement: Extended Data 1 — Statistical table. The statistical table provided contains all effect sizes, along with their 95% confidence intervals, and details regarding the methods of calculation. Download Extended Data 1, DOCX file. [file eneuro-11-ENEURO.0043-24.2024-s002.docx]

**Statistical Table**

|  | **Figure** | **Data structure** | **Type of test** | **p-values** | **Power**  **effect size [95% confidence interval]** |
| --- | --- | --- | --- | --- | --- |
| **a** | 3B | Non-normal distribution | Friedman test; Bonferroni’s *post hoc* test | 1.000  0.006  0.006  0.006  0.006  1.000 | Trial 2 vs 1: -0.531 [-1.153, 0.115]  Trial 14 vs 1: -1.986 [-3.012, -0.930]  Trial 15 vs 1: -2.266 [-3.394, -1.109]  Trial 14 vs 2: -2.181 [-3.277, -1.055]  Trial 15 vs 2: -2.638 [-3.907, -1.343]  Trial 15 vs 14: 0.351 [-0.267, 0.953] |
| **b** | 3C | Non-normal distribution | Friedman test; Bonferroni’s *post hoc* test | 0.239  0.002  0.002  0.002  0.002  1.000 | Trial 2 vs 1: -0.625 [-1.212, -0.017]  Trial 14 vs 1: -1.194 [-1.900, -0.460]  Trial 15 vs 1: -1.164 [-1.862, -0.438]  Trial 14 vs 2: -1.524 [-2.321, -0.698]  Trial 15 vs 2: -1.418 [-2.185, -0.623]  Trial 15 vs 14: -0.168 [-0.712, 0.383] |
| **c** | - | Non-normal distribution | Friedman test; Bonferroni’s *post hoc* test | 1.000  0.006  0.006  0.006  0.006  0.885 | Trial 2 vs 1 (TA): 0.205 [-0.397, 0.798]  Trial 14 vs 1 (TA): 1.957 [0.911, 2.972]  Trial 15 vs 1 (TA): 2.024 [0.954, 3.063]  Trial 14 vs 2 (TA): 2.014 [0.948, 3.050]  Trial 15 vs 2 (TA): 2.079 [0.990, 3.139]  Trial 15 vs 14 (TA): -0.620 [-1.256, 0.042] |
| **d** | - | Non-normal distribution | Friedman test; Bonferroni’s *post hoc* test | 0.129  0.002  0.002  0.002  0.002  1.000 | Trial 2 vs 1 (FDI): 0.664 [0.049, 1.256]  Trial 14 vs 1 (FDI): 1.896 [0.957, 2.808]  Trial 15 vs 1 (FDI): 1.962 [1.002, 2.896]  Trial 14 vs 2 (FDI): 1.426 [0.629, 2.195]  Trial 15 vs 2 (FDI): 1.547 [0.715, 2.352]  Trial 15 vs 14 (FDI): -0.065 [-0.608, 0.480] |
| **e** | 4B | Non-normal distribution | Wilcoxon signed-rank test | 0.005  < 0.001 | TA: -1.211 [-1.984, -0.405]  FDI: -0.902 [-1.539, -0.239] |
| **f** | 4D | Non-normal distribution | Wilcoxon signed-rank test | < 0.001  0.003 | Delta band: -1.388 [-2.213, -0.529]  Alpha band: -0.866 [-1.551, -0.151] |
| **g** | 4E | Non-normal distribution | Wilcoxon signed-rank test | < 0.001  0.002 | Delta band: -0.540 [-1.115, 0.053]  Alpha band: -0.586 [-1.167, 0.015] |
| **h** | 5A | Residuals normally distributed | Linear mixed models | < 0.001 | 0.578 [0.307, 0.849] |
| **i** | 5C | Residuals normally distributed | Linear mixed models | < 0.001 | -0.577 [-0.830, -0.325] |
| **j** | 5B | Residuals normally distributed | Linear mixed models | 0.025 | 0.408 [0.021, 0.795] |
| **k** | 5D | Residuals normally distributed | Linear mixed models | 0.003 | -0.537 [-0.917, -0.158] |
| **l** | 6C | Non-normal distribution | One-sample Wilcoxon signed-rank test | 0.557  0.014  0.322 | Delta band: -0.009 [-0.628, 0.611]  Alpha band: -1.188 [-1.992, -0.347]  Beta band: -0.453 [-1.095, 0.212] |
| **m** | 6D | Non-normal distribution | One-sample Wilcoxon signed-rank test | 0.910  0.008  0.074 | Delta band: 0.159 [-0.504, 0.812]  Alpha band: -1.130 [-1.960, -0.260]  Beta band: -0.689 [-1.406, 0.060] |
| **n** | 7A | Non-normal distribution | One-sample Wilcoxon signed-rank test | < 0.001  < 0.001 | TA: 1.913 [0.882, 2.913]  FDI: 1.503 [0.684, 2.295] |
| **o** | 7B | Non-normal distribution | One-sample Wilcoxon signed-rank test | 0.003  0.003 | TA: 1.275 [0.451, 2.067]  FDI: 0.863 [0.208, 1.492] |
| **p** | 8A | Residuals normally distributed | Repeated measures correlations | 0.008 | 0.574 [0.176, 0.811] |
| **Q** | 8B | Residuals normally distributed | Repeated measures correlations | 0.002 | -0.637 [-0.843, -0.273] |
| **r** | 8C | Residuals normally distributed | Repeated measures correlations | 0.004 | -0.619 [-0.833, -0.243] |

*effect sizes in lines* ***a, b, c, d, e, f, g*** *were calculated using Cohen’s dz (Difference score standard deviation) as reported in JANE et al. (2024)^1^.*

*effect sizes in lines* ***h, i, j, k*** *were calculated using the function ‘eff_size’ in R, which estimates Cohen’s d directly from the outputs of the functions ‘lm’ and ‘emmeans’^2^.*

*effect sizes in lines* ***l, m, n, o*** *were calculated using Cohen’s ds (Single Group) as reported in JANE et al. (2024)^1^.*

*effect sizes in lines* ***p, q, r*** *were calculated using the function ‘rmcorr’ in R, which already estimates correlation effect sizes.*

*TA, tibialis anterior; FDI, first dorsal interosseous.*

**References**

^1^Jané, M., Xiao, Q., Yeung, S., Ben-Shachar, M. S., Caldwell, A., Cousineau, D., Dunleavy, D. J., Elsherif, M., Johnson, B., Moreau, D., Riesthuis, P., Röseler, L., Steele, J., Vieira, F., Zloteanu, M., & Feldman, G. (2024). Guide to Effect Sizes and Confidence Intervals. <http://dx.doi.org/10.17605/OSF.IO/D8C4G>

^2^https://cran.r-project.org/web/packages/emmeans/vignettes/comparisons.html
